# Cancer cachexia associates with a systemic autophagy-inducing activity mimicked by cancer cell-derived IL-6 trans-signaling

**DOI:** 10.1038/s41598-017-02088-2

**Published:** 2017-05-17

**Authors:** Kristine Pettersen, Sonja Andersen, Simone Degen, Valentina Tadini, Joël Grosjean, Shinji Hatakeyama, Almaz N. Tesfahun, Siver Moestue, Jana Kim, Unni Nonstad, Pål R. Romundstad, Frank Skorpen, Sveinung Sørhaug, Tore Amundsen, Bjørn H. Grønberg, Florian Strasser, Nathan Stephens, Dag Hoem, Anders Molven, Stein Kaasa, Kenneth Fearon, Carsten Jacobi, Geir Bjørkøy

**Affiliations:** 1 Department of Medical Laboratory Technology, Faculty of Natural Sciences, NTNU - Norwegian University of Science and Technology, 7491 Trondheim, Norway; 2 Centre of Molecular Inflammation Research and Department of Cancer Research and Molecular Medicine, NTNU - Norwegian University of Science and Technology, 7030 Trondheim, Norway; 3 Musculoskeletal Disease Area, Novartis Institutes for BioMedical Research Basel, Novartis Pharma AG, 4056 Basel, Switzerland; 4 Department of Laboratory Medicine, Children’s and Women’s Health, Faculty of Medicine and Health Sciences, NTNU - Norwegian University of Science and Technology, 7491 Trondheim, Norway; 5 Department of Circulation and Medical Imaging, Faculty of Medicine and Health Sciences, NTNU - Norwegian University of Science and Technology, 7491 Trondheim, Norway; 6 Department of Public Health and General Practice, Faculty of Medicine and Health Sciences, NTNU - Norwegian University of Science and Technology, 7491 Trondheim, Norway; 7 European Palliative Care Research Centre, Department of Cancer Research and Molecular Medicine, Faculty of Medicine and Health Sciences, NTNU - Norwegian University of Science and Technology, 7030 Trondheim, Norway; 8 Department of Thoracic Medicine, St.Olavs Hospital - Trondheim University Hospital, 7006 Trondheim, Norway; 9 The Cancer Clinic, St.Olavs Hospital - Trondheim University Hospital, 7030 Trondheim, Norway; 10 Department of Cancer Research and Molecular Medicine, Faculty of Medicine and Health Sciences, NTNU-Norwegian University of Science and Technology, Trondheim, Norway; 11 Oncological Palliative Medicine, Division ofClinic Oncology/Hematology, Department of Internal Medicine and Palliative Care Center, Cantonal Hospital, St. Gallen, Switzerland; 12 Clinical and Surgical Sciences, School of Clinical Sciences and Community Health, The University of Edinburgh, Royal Infirmary, N-5021 Edinburgh, UK; 13 Department of Gastrointestinal Surgery, Haukeland University Hospital, N-5020 Bergen, Norway; 14 Gade Laboratory for Pathology, Department of Clinical Medicine, University of Bergen, N-5021 Bergen, Norway

## Abstract

The majority of cancer patients with advanced disease experience weight loss, including loss of lean body mass. Severe weight loss is characteristic for cancer cachexia, a condition that significantly impairs functional status and survival. The underlying causes of cachexia are incompletely understood, and currently no therapeutic approach can completely reverse the condition. Autophagy coordinates lysosomal destruction of cytosolic constituents and is systemically induced by starvation. We hypothesized that starvation-mimicking signaling compounds secreted from tumor cells may cause a systemic acceleration of autophagy during cachexia. We found that IL-6 secreted by tumor cells accelerates autophagy in myotubes when complexed with soluble IL-6 receptor (trans-signaling). In lung cancer patients, were cachexia is prevalent, there was a significant correlation between elevated IL-6 expression in the tumor and poor prognosis of the patients. We found evidence for an autophagy-inducing bioactivity in serum from cancer patients and that this is clearly associated with weight loss. Importantly, the autophagy-inducing bioactivity was reduced by interference with IL-6 trans-signaling. Together, our findings suggest that IL-6 trans-signaling may be targeted in cancer cachexia.

## Introduction

Between 60 and 80% of cancer patients develop cachexia^1^, a condition characterized by massive loss of lean body mass (with or without loss of fat mass). The condition often comprises functional impairment, reduced quality of life, increased risk of cancer treatment failure and significantly impaired survival^2^. Of cancer patients, 10–30% are believed to die from cachexia, the prevalence varying between cancer types^1^. Currently, no therapeutic approach can completely reverse the condition. It is therefore necessary to unravel key underlying factors or processes that may be targeted in cachexia therapy to improve life quality and prolong survival of cancer patients.

Several causative factors for cachexia have been suggested. Increased levels of circulating pro-inflammatory cytokines, such as interleukin 6 (IL-6), tumor necrosis factor α (TNFα) and interferon γ (IFNγ) as well as zinc-α2-glycoprotein (ZAG), proteolysis-inducing factor (PIF) and activin A have been suggested to correlate with the condition^3^. Some studies also link tumor-derived parathyroid-hormone related protein (PTHrP) to energy wasting in both adipose and muscle tissue^4^. Excessive catabolism is thought to play a major role in the development of cachexia^5^ and factors, such as those mentioned above, may trigger an increased intracellular degradation.

Intracellular protein degradation occurs in proteasomes and lysosomes. Markers of increased proteasomal degradation, such as atrogin-1/MAFbx and MuRF-1, are detected in some groups of cachectic patients and may contribute to muscle loss^6, 7^. Macroautophagy (hereafter referred to as autophagy) directs cytoplasmic constituents to lysosomal degradation. A possible role of elevated autophagy in cachexia development has recently emerged^8–12^. The process involves the sequestration of cytoplasm into double-membrane vesicles, autophagosomes, which fuse with lysosomes, thereby degrading the content. Autophagy can be highly selective and is strictly regulated. All cells have a basal autophagy flux, meaning that cellular content is degraded at a basal speed by autophagy. However, the autophagy flux can be accelerated or inhibited by different stimuli, thereby altering the turn-over time of cellular content^13^. Starvation causes a strong inducing of autophagy and the process mobilizes nutrients and essential amino acids^14, 15^. Survival of mice depends on functional autophagy, both during low nutrient availability, such as that experienced shortly after birth (neonatal phase)^14^, and acute starvation of adults^15^. This highlights that under certain circumstances, autophagy may be induced systemically. However, it is currently not fully understood how systemic autophagy is coordinated and regulated.

Tumor growth is associated with reduced availability of nutrients. Tumor cells therefore make certain adaptations to increase nutrient supply and sustain survival and proliferation^16^. It has been suggested that cancer cells secrete signaling substances that can accelerate autophagy in other cells in the tumor micro-environment^17, 18^. The nutrients that are generated and released following increased autophagy may benefit cancer cells and sustain tumor growth. It is not known whether such cellular cross talk occurs only locally within the tumor or whether a systemic variant exists.

We hypothesized that cancer cachexia involves systemic acceleration of autophagy induced by starvation-mimicking signaling compounds secreted from tumor cells. We found that cancer cells with the ability to accelerate autophagy in cell cultures also caused cachexia as xenografts in mice. Conditioned medium from the cachexia-inducing cancer cells contained high levels of IL-6 and neutralizing this cytokine strongly reduced the autophagy-inducing activity. Moreover, IL-6 was a potent inducer of autophagy in myotubes when bound to soluble IL-6 receptor in a complex that can stimulate signaling via the gp130 receptor (trans-signaling). Consistent with an important role of IL-6 in inducing cachexia, there is an association between elevated tumor specific expression of IL-6 and poor prognosis of lung cancer patients where the prevalence of fatal cachexia is high. Furthermore, we found that autophagy-inducing bioactivity in serum was significantly associated with weight loss in lung and gastrointestinal cancer patients. This bioactivity was reduced when IL-6 trans-signaling was inhibited by soluble gp130Fc. Together, we show that IL-6 trans-signaling is a novel autophagy-inducing pathway that may be important in cachexia development and targeted in cachectic patients.

## Results

### Sera from cancer patients with weight loss contain autophagy-inducing bioactivity

**Figure 1:**
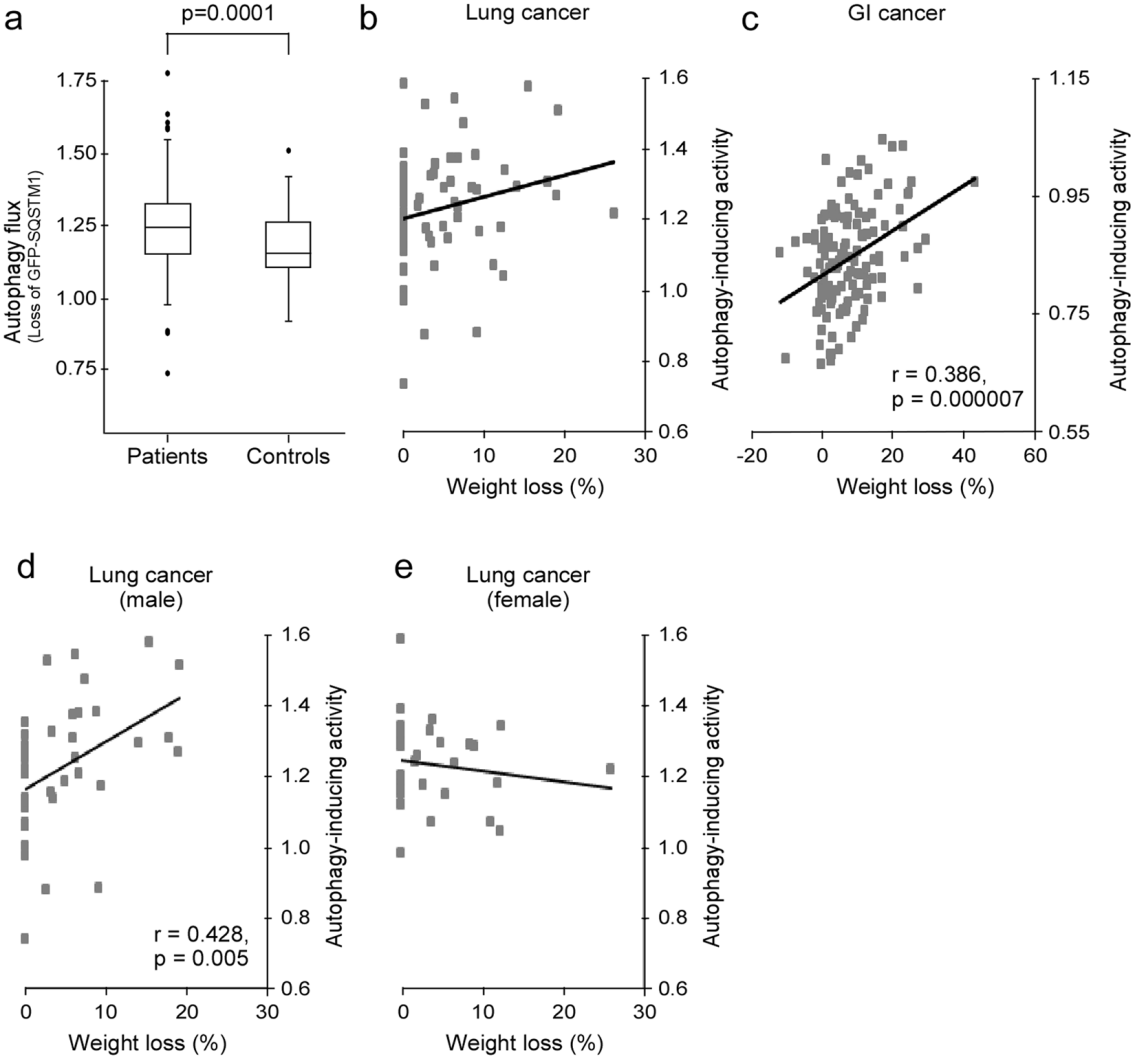
Blood samples from cancer patients with weight loss harbor autophagy-inducing bioactivity. (**a**) Autophagy flux in reporter cells exposed to sera from lung cancer patients (n = 79) or healthy controls (n = 148) (Student t-test). (**b**) Weight loss (% loss during 3 months prior to blood sampling) in lung cancer patients and autophagy-inducing activity in their sera, measured using an autophagy reporter cells system. (**c**) Weight loss (% loss during 6 months prior to blood sampling) in GI cancer patients and autophagy-inducing activity in their plasma, measured using an autophagy reporter cells system. (**d**,**e)** As in (**b**), but divided by gender.

We hypothesized that cancer cachexia involves systemic acceleration of autophagy. For autophagy to be accelerated systemically, autophagy-inducing bioactivity must be present in circulation. Sera from 79 patients from The Central Norway Lung Cancer Biobank (CNLCB), collected at the time of diagnosis, and 148 healthy controls were tested for autophagy-inducing bioactivity, using a cell based autophagy quantification assay (hereafter referred to as autophagy reporter cells)^19^. Briefly, the assay is based on detecting the degradation rate of the autophagy marker, SQSTM1, fused to green fluorescent protein (GFP), by flow cytometry. We found that sera from the lung cancer patients gave a higher autophagy flux in the reporter cells compared with control sera (Fig. 1a). When defining accelerated autophagy as more than 1 standard deviation (SD) higher than the control group mean, we also found that a higher proportion of the sera from lung cancer patients accelerated autophagy (38%), compared with the controls (17%). The different ability to accelerate autophagy was not due to differences in age, gender and smoking habit as the groups were matched for these variables. All patient sera were collected at the time of diagnosis and thus before initiation of cancer treatment. Therefore, autophagy-accelerating bioactivity was not treatment related.

Next we investigated the possible association between autophagy-inducing bioactivity and weight loss of the patients, a hallmark of cachexia. We observed that the patients providing sera that induced autophagy were more likely to have experienced a weight loss (>5% loss of body mass during the last three months prior to diagnosis) as compared with those whose sera did not (Pearson chi-square, n = 75, p = 0.025) (Fig. 1b). Even though weight loss was more likely to occur for patients whose sera induced autophagy, no significant linear correlation between autophagy-inducing bioactivity and weight loss was detected (r = 0.22, p = 0.62). In a second cohort of 127 plasma samples, however, from patients with gastrointestinal cancer included in the Edinburgh Cancer Cachexia Cohort (ECCC) study we found a stronger association between autophagy-inducing activity and weight loss: for each 50% increase in autophagy, the estimated weight loss was increased by 20% (Pearson chi-square, p = 0.000007, 95% CI 12–28%) (Fig. 1c). Notably, both lung and gastrointestinal cancer patients frequently develop cachexia^20^.

We also analyzed samples from a third, more heterogeneous cohort (European Pharmacogenetic Opioid Study [EPOS]) of cancer patients with advanced disease. This cohort included many hematological and breast cancer patients, where cachexia is less frequent^20^. Interestingly, in this cohort, we found no significant association between weight loss and autophagy-inducing ability of sera (Pearson chi-square, n = 75, p = 0.51). Together, this suggests that the association between autophagy acceleration and weight loss is dependent on tumor type and prevalence of cachexia.

It has previously been suggested that there may be sexual dimorphisms in cancer cachexia^21^, with men losing weight faster and surviving shorter (at least in lung cancer) than women^22^. Interestingly, we found a clear association between weight loss and autophagy-inducing ability of the sera from the male lung cancer patients (n = 42): for each 50% increase in autophagy the estimated weight loss was increased by 7% (Pearson chi-square, p = 0.005, 95% CI 2–11%) (Fig. 1d). For the female lung cancer patients (n = 33) there was no association between weight loss and autophagy-inducing ability (Fig. 1d). Among the GI cancer patients, weight loss and autophagy-inducing capability were associated for both genders. This indicates that there might be sexual dimorphic and tumor type-specific aspects involved in the regulation of autophagy during cancer cachexia.

### Cancer cells that cause cachexia secrete factors that induce autophagy

**Figure 2:**
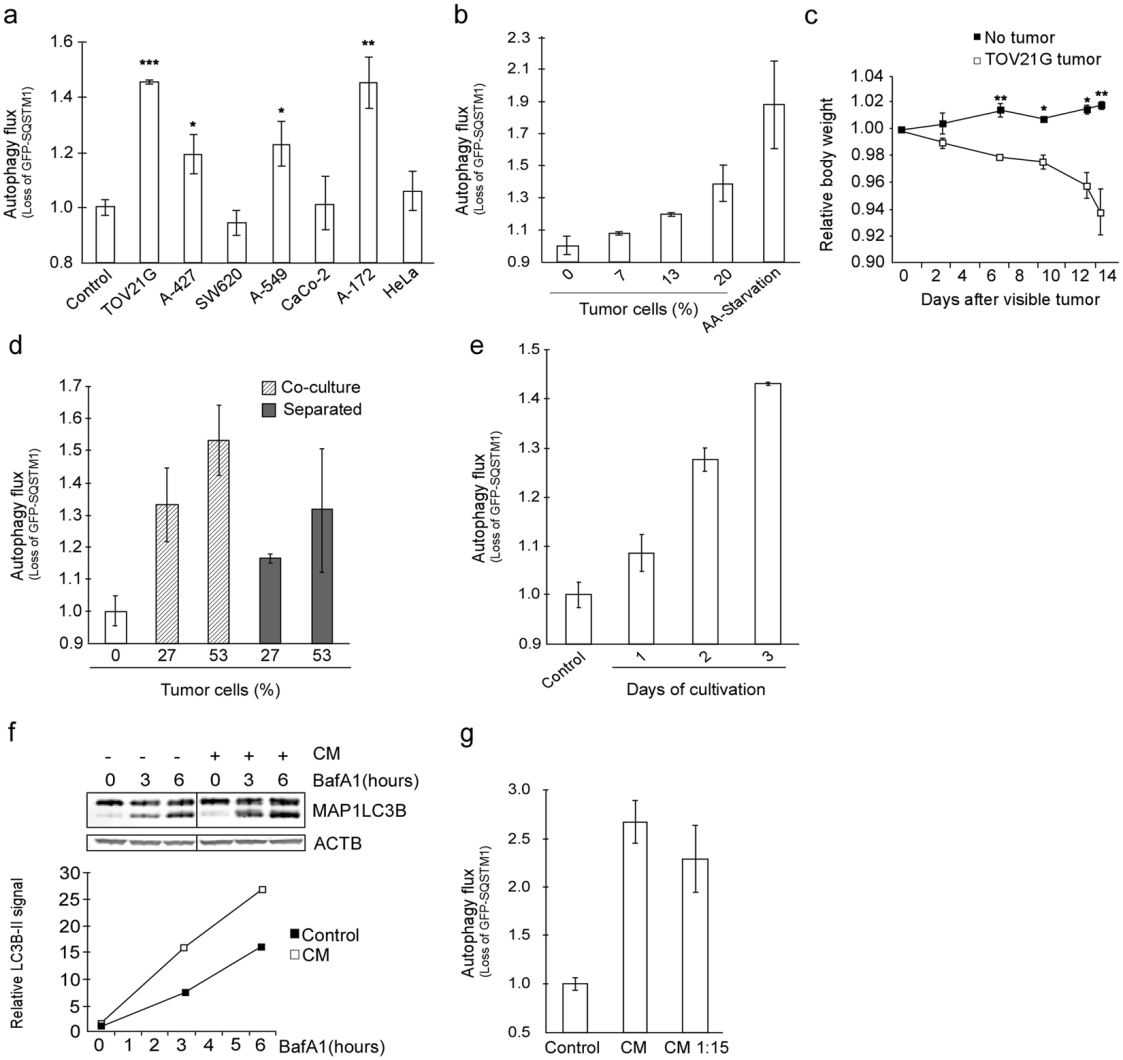
TOV21G cancer cells cause cachexia and secrete autophagy-inducing substances. (**a**) Screen of various cancer cell lines for the ability to induce autophagy in reporter cells (co-cultures). Mean from one experiment using triplicate wells ± SD. *p = 0.04, **p = 0.02, ***p = 0.008 vs control (Student t-test). (**b**) Reporter cells co-cultured with increasing percentage of TOV21G cells. Amino acid starvation (HBSS, 16 h) as positive control. Mean from one experiment representative of six experiments using duplicate wells ± SD. (**c**) Mean relative body weight (at indicated time intervals) of TOV21G tumor-bearing mice (n = 6) and control mice (n = 6) ± SEM. *p < 0.01, **p < 0.005 vs no tumor (Student t-test). (**d**) Autophagy flux in reporter cells co-cultured with or separated from TOV21G cells using a semi-permeable membrane. Mean from one experiment using duplicate wells ± SD. (**e**) Autophagy flux in reporter cells after 3 days of exposure to conditioned medium (CM) from TOV21G cells cultured for 1–3 days (n = 1 for 1 day and n > 6 for 3 days). Mean from one representative experiment using duplicate wells ± SD. (**f**) Protein levels of MAP1LC3B in reporter cells treated with CM from TOV21G cells for 3 days with or without BafA1 (100 nM) for indicated time periods. MAPLC3B-II signal is normalized against β-actin/ACTB. The data are representative for two independent experiments. Image, originating from the same blot, is cropped. (**g**) Autophagy flux in reporter cells exposed to diluted (1:15 in complete growth medium) or undiluted CM from TOV21G cells. Mean from one representative of three independent experiments using triplicate wells ± SD.

Sera from cancer patients with weight loss contain autophagy-inducing bioactivity. Due to the limited availability of patient sera we utilized cell based methods to identify the nature of this bioactivity. Cancer cachexia is initiated by the tumor and systemic autophagy-accelerating bioactivity may be attributed to factor(s) derived from the cancer cells. To find a suitable working model we first screened cancer cells using the autophagy reporter cell assay. When co-culturing the reporter cells with seven cancer cell lines from five cancer forms, we found that the cancer cell lines displayed very different ability to induce autophagy in the reporter cells (Fig. 2a). The ovarian carcinoma TOV21G cell line was among the most potent autophagy-inducers. Autophagy increased with number of TOV21G cells and even when these cancer cells comprised only 20% of the total number of cells in the co-culture, they induced autophagy to a level approximately half of that obtained by complete amino acid starvation (Fig. 2b). As previously reported^23^, mice with TOV21G tumors rapidly lost weight (Fig. 2c).

Together, these results indicated that TOV21G cells secrete compounds which may increase autophagy systemically and thereby contribute to cachexia. Consistently, separating the TOV21G cells and the autophagy reporter cells by a semi-permeable membrane or employing a conditioned medium (CM) approach did not eradicate the cancer cell-induced autophagy in the reporter cells. This confirmed that autophagy acceleration was not dependent on cell-to-cell contact (Fig. 2d,e). CM from the cancer cells also accelerated autophagy more potently with prolonged time of cultivation (Fig. 2e). Consistently, the CM induced autophagy also when determined as MAP1LC3B-II accumulation quantified by immunoblotting (Fig. 2f).

Nutrient restriction is a potent autophagy accelerator. However, even when diluted 1:15 in complete, fresh medium, the ability of CM to accelerate autophagy was not reduced (Fig. 2g). This demonstrates that the autophagy induction in the reporter cells is caused by one or more secreted factors and not a lack of nutrients.

### IL-6 secreted from cancer cells acts as an inducer of autophagy

**Figure 3:**
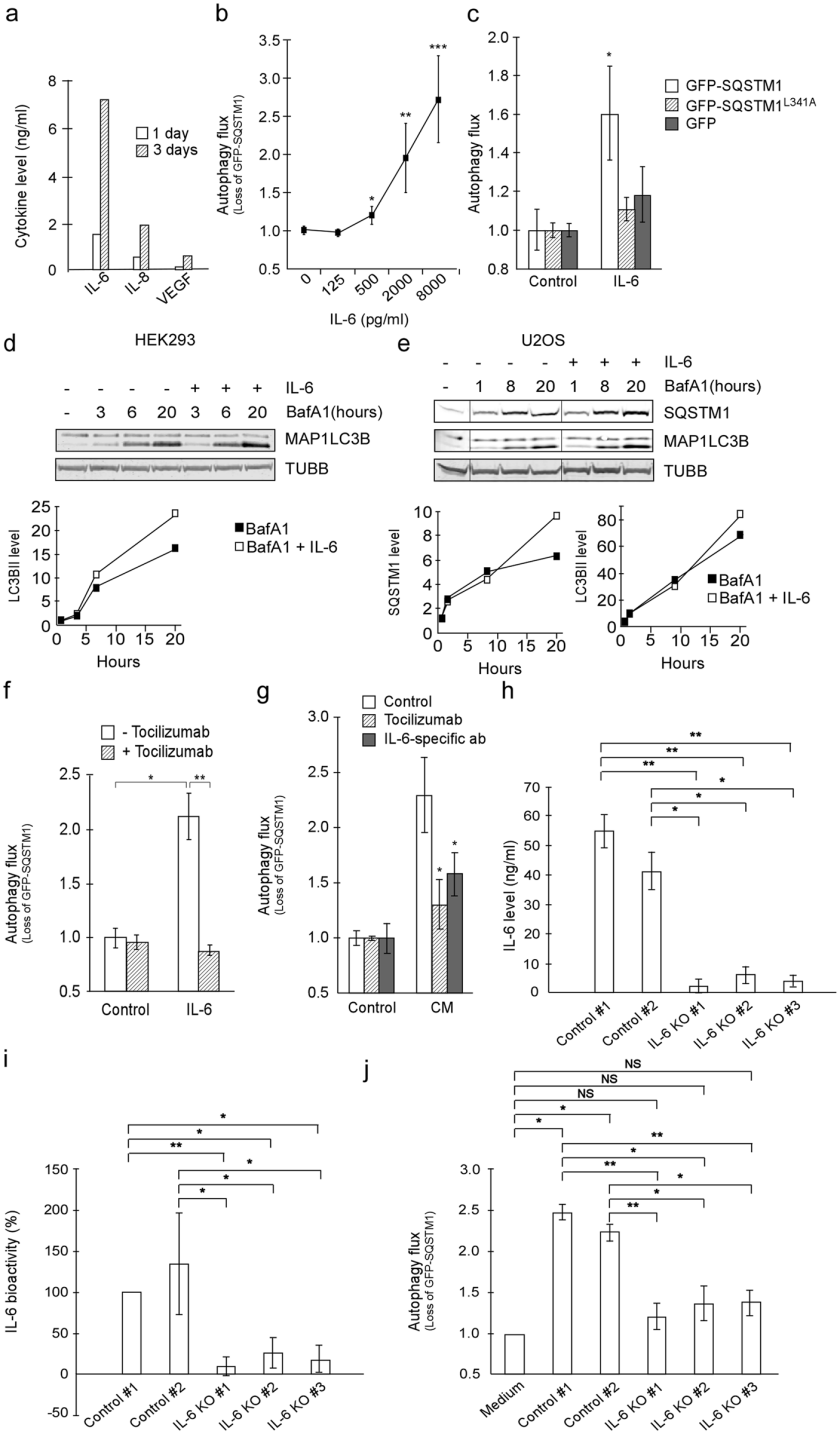
IL-6 secreted from TOV21G cells induces autophagy. (**a**) Bio-Plex assay of TOV21G conditioned medium (CM) (1 and 3 days cultivation). (**b**) Autophagy flux in reporter cells following 3 days of rIL-6 exposure. Mean from three independent experiments using duplicate wells ± SD. *p < 0.005, **p < 1 × 10^−4^, ***p < 5 × 10^−6^ vs control (Student t-test). (**c**) Degradation rate of GFP-SQSTM1, GFP and GFP-SQSTM1^L341A^ protein in HEK293 cells following rIL-6 (20 ng/ml) exposure. Mean from one representative of three independent experiments using triplicate wells ± SD. *P = 0.01 vs control (Student t-test). (**d**) MAP1LC3B protein levels in reporter cells treated with rIL-6 (20 ng/ml) and/or BafA1 (100 nM). MAP1LC3B-II signals normalized against β-tubulin/TUBB. Data representative for five independent experiments. (**e**) As D, but U2OS cells stained for both MAP1LC3B and SQSTM1. Data representative for two independent experiments. Image, originating from the same blot, is cropped. (**f**) Autophagy flux in reporter cells treated with rIL-6 (20 ng/ml) alone or in combination with tocilizumab (including 30–60 minutes pretreatment, 10 µg/ml). Mean from one representative of three independent experiments using triplicate wells ± SD. *p < 0.005, **p < 0.0005 (Student t-test). (**g**) Autophagy flux in reporter cells following exposure to TOV21G CM (diluted 1:15 in complete growth medium) w/wo tocilizumab (30–60 minutes pretreatment, 10 µg/ml) or IL-6-specific ab (30–60 minutes pre-incubation of CM, 3 µg/ml). Mean from one representative of three independent experiments using triplicate wells ± SD. *p < 0.05 vs CM, control (Student t-test). (**h**) IL-6 level in CM from TOV21G cells without (Controls #1 and #2) and with (IL-6 KO #1–3) IL-6 knock down measured by ELISA assay. Mean from three independent experiments using triplicate wells ± SD. *p < 0.005, **p < 0.0005 (Student t-test). **I)** Relative IL-6 bioactivity in CM from TOV21G cells (diluted 1:2 in fresh growth medium) w/wo IL-6 knock down measured by a B9 cell-based IL-6 bioactivity assay. Mean from three independent experiments using triplicate wells ± SD. *p < 0.05, **p < 0.01 (Student t-test). **J)** Autophagy flux in reporter cells following exposure to CM (diluted 1:15 in complete growth medium) from TOV21G cells w/wo IL-6 knock down. Mean from three independent experiments using triplicate wells ± SD. NS = not significant, *p < 0.05, **p < 0.005, ***p < 0.0005 (Student t-test).

To search for secreted compounds from the TOV21G cells, CM was analyzed using a multiplex setup of 27 cytokines, chemokines and growth factors (Supplementary Table S1). High levels of three factors were identified; IL-6, IL-8, and VEGF, and their concentration increased by time (Fig. 3a). Recombinant IL-6, IL-8, and VEGF all induced signaling in the autophagy reporter cells (Supplementary Figure S1), but only IL-6 caused a clear reduction in the level of GFP-SQSTM1 fusion protein (Fig. 3b, and data not shown). The decreased level of fusion protein corresponds to increased autophagy speed and not to reduced protein synthesis since the turnover of GFP or GFP-SQSTM1^L341A^ (a mutant not specifically targeted by autophagy) is not significantly affected by IL-6 (Fig. 3c). Addition of IL-6 also caused an increased turnover of MAP1LC3B-II in both HEK293 and U2OS cells when evaluated by immunoblot (Fig. 3d,e). The autophagy-inducing activity of the CM was reduced by adding neutralizing antibodies towards the IL-6 receptor or IL-6 (Fig. 3f,g). Consistently, knock down of IL-6 in TOV21G (Fig. 3h,i) reduced the autophagy-inducing activity of CM (Fig. 3j). Of note, knock down of IL-6 did not cause any clear difference in growth rate of the cancer cells (data not shown). To summarize, IL-6 is secreted by TOV21G cancer cells, accelerates autophagy in other cells and may therefore be important in cachexia development.

### Cachexia-causing cancer cells accelerate autophagy

**Figure 4:**
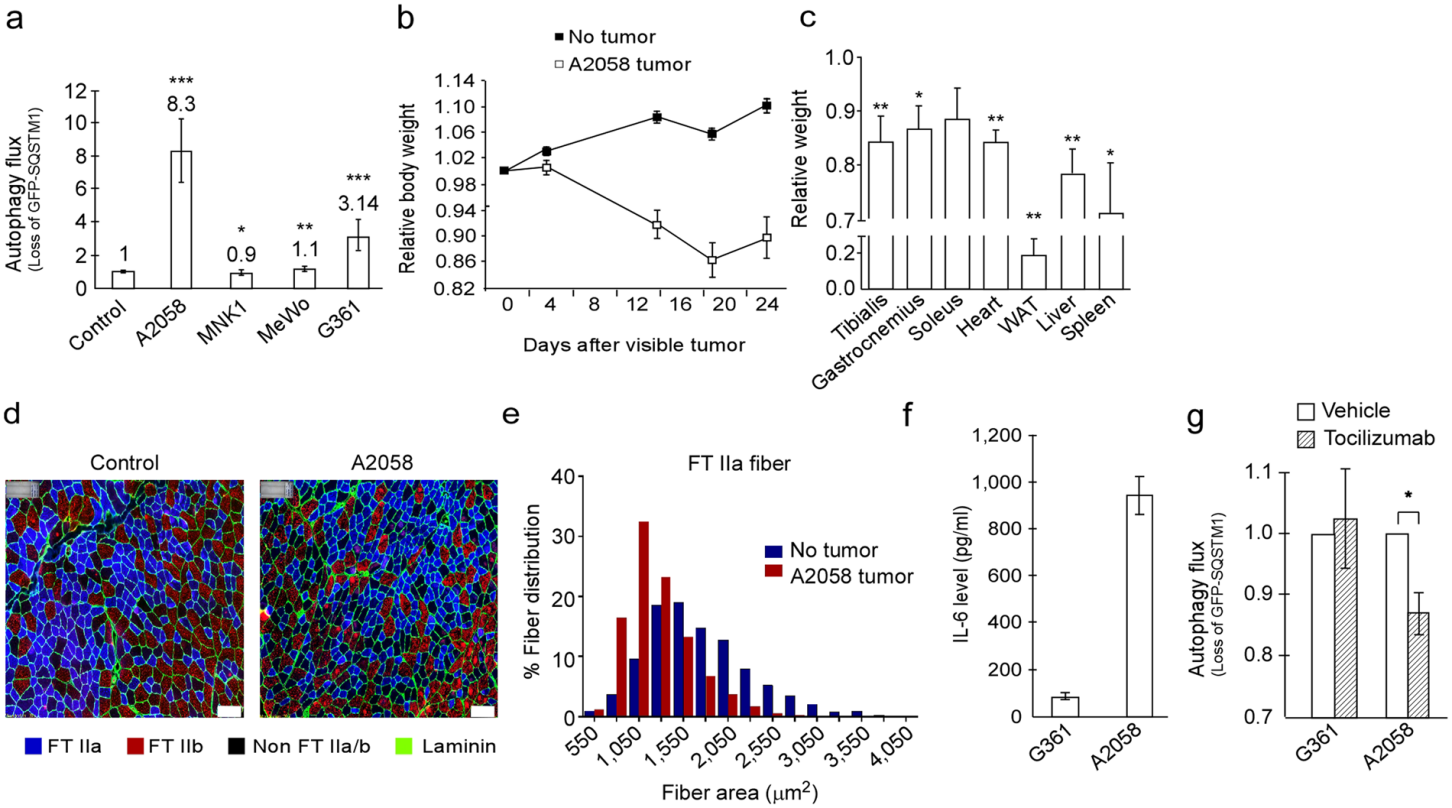
Autophagy-inducing cancer cells cause cachexia in mice. (**a**) Autophagy flux in reporter cells exposed to conditioned medium from various cancer cell lines. Mean autophagy flux from four experiments using triplicate wells ± SD. Fold change compared to control is indicated. *p < 0.05, **p < 0.005, ***p < 2 × 10^−8^ vs control (Student t-test). (**b**) Mean relative body weight (at indicated time intervals) ± SEM of A2058 tumor-bearing mice (n = 10) and no tumor control mice (n = 10). (**c**) Mean muscle, white adipose tissue (WAT) and organ weight ± SEM of A2058 tumor-bearing mice (n = 10) relative to no tumor control mice (n = 10). *p < 0.05, **p < 0.01 vs no tumor (Student t-test). (**d**) Cross section of tibialis muscle from control and A2058 tumor-bearing mice. Immunostaining of Fast Twitch (FT) IIa, IIb muscle fibers and laminin as indicated. Scale bar = 100 µm. (**e**) Mean tibialis muscle FT IIa fiber area in control (n = 3) and A2058 tumor-bearing mice (n = 4). (**f**) IL-6 ELISA assay of conditioned medium from the indicated cell lines. Mean from three (G361) or four (A2058) independent experiments using triplicate wells ± SD. (**g**) Autophagy flux in reporter cells following exposure to conditioned medium from the indicated cell lines (diluted 1:15 in complete growth medium) with or without tocilizumab (30–60 minutes pretreatment, 10 µg/ml). Mean from three (A2058) or four (G361) independent experiments using triplicate wells ± SD. *p < 0.05 (Student t-test).

Having found that TOV21G cells cause cachexia in mice and can accelerate autophagy *in vitro*, we aimed to determine whether these abilities coincided also for other cancer cells. CMs from four cancer cell lines were analyzed for autophagy-inducing potential *in vitro* (Fig. 4a). Two CMs (from MKN1 and MEWO) were unable to or only marginally accelerated autophagy in the reporter cells. In xenograft mouse models, MEWO tumors did not cause any weight loss^24^ and MKN1 tumors only caused weight loss at an advanced stage (following day 30 after tumor initiation) (Supplementary Figure S2). CMs from the remaining two cell lines, A2058 and G361, accelerated autophagy. Because the ability of both these cancer cell lines to induce autophagy was monitored in the presence of nutrient-rich growth medium (with 10% serum), the detected autophagy acceleration was not a starvation response. Importantly, tumors formed by A2058 and G361 caused a significant loss of weight in xenograft mouse models (Fig. 4b and Supplementary Figure S2). In addition, muscle, white adipose tissue (WAT) and organ weight loss was observed, as well as a switch to smaller muscle fibers (Fast Twitch IIa and IIb) (Fig. 4c–e, Supplementary Figure S2 and Supplementary Table S2). Together, these results indicate that cancer cells capable of causing cachexia secrete substances that increase catabolism and induce atrophy in different tissues, including muscle. It is however, to be noted that, *in vivo*, the autophagy-inducing factor(s) from the cancer cells could be accompanied by other cancer-associated conditions to cause cachexia. Although all the mice in these experiments had food and water freely accessible (ad libitum feeding) one of the co-factors to cause cachexia could be reduced food intake, resulting in semi-starvation, not measured in the present study.

When analyzing CMs from these cells lines for IL-6 we found that all cell lines secreted the cytokine, however, A2058 secreted substantially higher amounts of IL-6 than G361 cells (Fig. 4f). Importantly, the IL-6 receptor neutralizing antibody, tocilizumab significantly reduces the autophagy-accelerating effect of A2058 CM (Fig. 4g). This is in support of an important role of IL-6 in autophagy acceleration and cachexia development. The amount of IL-6 secreted from G361 cells was well below the threshold level to cause an autophagy-inducing response in the HEK293-based bioassay (Fig. 3b). Accordingly, tocilizumab was not able to reduce the autophagy-accelerating effect of CM from G361 (Fig. 4g). This finding is not surprising since it is likely that diverse factors are secreted from cancer cells and involved in stimulation of cachexia.

### IL-6 trans-signaling accelerates autophagy in muscle

Although we demonstrate here that IL-6 directly can affect autophagy, it is important to consider that most cell types, including muscle cells, have a generally low expression of IL-6 receptor^25^. These cells may therefore not respond potently to classical IL-6 signaling (cis-signaling) which is initiated when IL-6 binds to a transmembrane IL-6 receptor on the target cell. However, IL-6 may also act by an alternative trans-signaling mechanism, when IL-6 complexed to soluble IL-6 receptor (sIL-6R) binds to the transmembrane co-receptor gp130.

**Figure 5:**
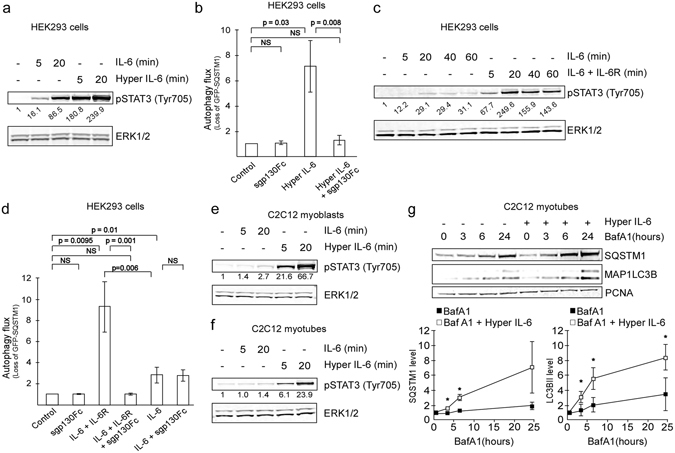
IL-6 trans-signaling accelerates autophagy in myotubes. (**a**) Protein levels of pSTAT3^Tyr705^ in HEK293 reporter cells treated with rIL-6 (20 ng/ml) or conditioned medium from hyper IL-6-producing CHO cells (0.1%) as indicated. Protein level from one experiment relative to control and normalized against ERK1/2 as indicated. Results are representative of two independent experiments. (**b**) Autophagy flux in reporter cells following exposure to conditioned medium from hyper IL-6-producing CHO cells (0.1%) and/or sgp130Fc (1 µg/ml) as indicated. Mean from three independent experiments using duplicate (n = 1) or triplicate (n = 2) wells ± SD. NS = not significant (Student t-test). (**c**) Protein levels of pSTAT3^Tyr705^ in HEK293 reporter cells treated with rIL-6 (8 ng/ml) alone or in combination with rIL-6 receptor alpha (IL-6R, 50× molar excess relative to IL-6) as indicated. Protein level relative to control and normalized against ERK1/2 as indicated. (**d**) Autophagy flux in reporter cells following exposure to rIL-6 (8 ng/ml) and rIL-6 receptor alpha (IL-6R, 50× molar excess relative to IL-6) and/or sgp130Fc (1 µg/ml) as indicated. Mean from four (three for sgp130Fc) independent experiments using quadruplicate wells ± SD. NS = not significant (Student t-test). (**e**) Protein levels of pSTAT3^Tyr705^ in C2C12 myoblasts treated with rIL-6 (20 ng/ml) or conditioned medium from hyper IL-6-producing CHO cells (0.1%) as indicated. Protein level relative to control and normalized against ERK1/2 as indicated. (**f**) As E, but with C2C12 myotubes. (**g**) Protein levels of MAP1LC3B and SQSTM1 in C2C12 myotubes treated with conditioned medium from hyper IL-6-producing CHO cells (0.1%, 3 days) and BafA1 (100 nM) or BafA1 alone for indicated time periods. MAP1LC3B-II and SQSTM1 signals are normalized against PCNA. Graphs illustrates the mean from three independent experiments ± SD. *p < 0.05 (Student t-test).

To investigate the potential role of trans-signaling in acceleration of autophagy we subjected autophagy-reporter cells to a recombinant fusion-protein construct of IL-6 and sIL-6R (hyper IL-6). Hyper IL-6 induced signaling in the reporter cells (Fig. 5a) and potently accelerated autophagy (Fig. 5b). In accordance, combining recombinant IL-6 and sIL-6R induced signaling and caused a robust induction of autophagy (Fig. 5c,d). IL-6 in combination with sIL-6R much more potently induced autophagy than IL-6 alone (Fig. 5d). As expected, autophagy acceleration caused by hyper IL-6, as well as recombinant IL-6/sIL-6R, was completely blocked by a soluble variant of gp130 (sgp130Fc) (Fig. 5b and d). Of note, sgp130Fc specifically inhibited IL-6 trans-signaling as autophagy acceleration caused by IL-6 was not affected by sgp130Fc (Fig. 5d). Given the low abundance of IL-6R on muscle cells, it is possible that, during cachexia, autophagy induced by IL-6 trans-signaling plays a dominant role. In accordance, we observed only a modest increase in STAT3 phosphorylation and no induction of autophagy in C2C12 myoblasts or myotubes in response to recombinant IL-6 (Fig. 5e,f and data not shown). However, we found that IL-6 trans-signaling strongly induced STAT3 phosphorylation and accelerated autophagy in C2C12 myotubes (Fig. 5f,g). Interestingly, the ability to induce autophagy was specific for differentiated tubes as no change in autophagy was observed in C2C12 myoblasts in response to hyper IL-6 (data not shown). The lack of autophagy-acceleration in myoblasts was not due to the inability of hyper IL-6 to induce signaling as we could measure a potent phosphorylation of STAT3 in response to the treatment (Fig. 5e). Taken together, IL-6 trans-signaling induce autophagy in differentiated muscle cells, and may therefore be relevant for the muscle loss experienced by cachectic patients.

### IL-6 signaling in non-tumor tissue may limit survival of lung cancer patients

**Figure 6:**
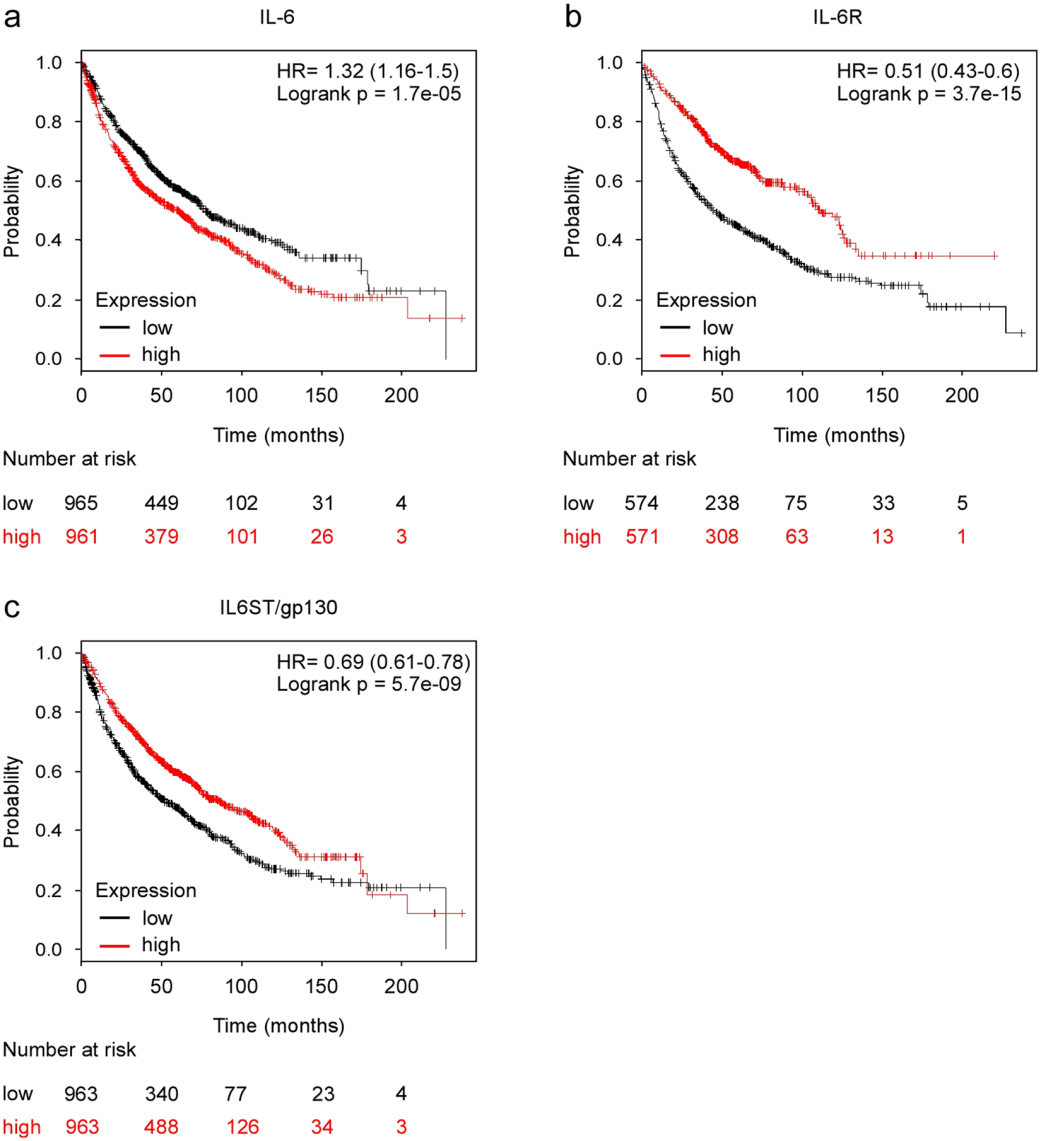
High expression of IL-6 mRNA in tumor tissue predicts poor prognosis of lung cancer patients. (**a**) High expression of IL-6 in the tumor predicts a worse overall survival of lung cancer patients. (**b**,**c**) High expression of IL-6 receptor and IL6ST/gp130, respectively, in the tumor predict prolonged survival of lung cancer patients. HR = hazard ratio. The data are obtained using the kmplot.com database tool^27, 28^.

Of the major tumor types, cachexia is prevalent in lung cancer patients^20^ and an independent predictor of short survival in this group of patients^26^. Since our data points to a possible mechanism for how cancer cells can accelerate autophagy (i.e. IL-6 secreted from cancer cells induces autophagy via trans-signaling), we asked if an increase in IL6 mRNA in the tumor correlates with short survival of lung cancer patients. The web based database kmplot^27, 28^ was used and the patients were split in two groups based on mRNA expression level in their tumor biopsies. This analysis demonstrated that there is a significantly shorter overall survival for lung cancer patients with IL-6 mRNA levels above the median value (HR = 1.32, longrank p = 1.7e-05, n = 1926) (Fig. 6a). Interestingly, intra-tumoral expression levels of both IL-6 receptor and the co-receptor IL6ST (gp130) are strong predictors of prolonged overall survival (Fig. 6b,c). These data indicate that IL-6 may induce detrimental responses outside the tumor in lung cancer patients. Of note, also the expression levels of other putative tumor-derived cachexia-inducing signaling compounds (OSM, LIF, TNFα, IFNγ, PTHrP, ZAG, PIF, TWEAK, IL-1β, MSTN and INHBA) were tested for possible correlation between expression level and prognosis for lung cancer patients. Only the expression level of IFNγ and PTHrP predicted poor prognosis but the association was far weaker than for IL6 (Supplementary Figure S3). Taken together, our findings show that IL-6 trans-signaling strongly accelerates autophagy in several cell types, including muscle, and may contribute to the pathogenesis of cachexia and survival of cancer patients.

Having found that IL-6 induces autophagy via trans-signaling and that tumor IL-6 mRNA expression is a predictor of poor prognosis in lung cancer patients we asked if there is an association between IL-6 levels and the autophagy inducing activity of the CNLCB lung cancer patient sera. Consistent with previous reports^29, 30^, the serum levels of IL-6 are low in the cancer patient sera (ranging from 0.6 pg/ml to 29.3 pg/ml [Supplementary Table S3]), but associated with CRP levels; for every 10 pg/ml increase in IL-6 the estimated increase in CRP was 0.92 mg/L (Pearson chi-square, p = 9.1e-9, n = 45, 95% CI 0.66–1.2 mg/L). Even though the differences in IL-6 serum levels were minor it could still be observed an association with weight loss; for each 10 pg/ml increase in IL-6 level there was an estimated 3.5% increase in weight loss (Pearson chi-square, p = 0.02, n = 46, 95% CI 0.5–6.5). IL-6-induced weight loss is probably not due to cis-signaling as we cannot detect IL-6 levels beyond the buffer capacity of soluble IL-6 receptors in circulation. Thus, in terms of weight loss, it is possible that IL-6 trans-signaling is the relevant mode of action. We find that IL-6 trans-signaling can accelerate autophagy in muscle cells. Additionally, we find an association between autophagy-accelerating bioactivity of cancer patient sera and weight loss of the patient. Together, this suggests that IL-6 trans-signaling-induced autophagy might be the relevant mechanism to cause weight loss. To more specifically test the importance of IL-6 trans-signaling in autophagy acceleration caused by lung cancer patient sera (CNLCB), soluble gp130Fc was applied to inhibit this mode of signaling. In line with an important role of IL-6 trans-signaling, autophagy acceleration in response to lung cancer patient sera was significantly reduced when trans-signaling was inhibited by soluble gp130Fc (Student t-test, p = 3.11e-05, 8% reduced autophagy activity, n = 7). Of note, sgp130Fc does not give a general reduction in autophagy (Fig. 5d). This suggests that IL-6 trans-signaling might be a relevant target to halt the development of cancer cachexia.

## Discussion

Cancer cachexia is a multifactorial condition affecting the majority of patients with advanced cancer. Both decreased protein synthesis and increased protein degradation likely contribute to the muscle wasting associated with cachexia. It is largely unknown which of these processes predominates and how these events are regulated^3^. Recently, we have shown that habitual myofibrillar protein synthesis is normal in weight-losing cancer patients, thereby suggesting that increased protein degradation may be the dominant process in humans^31^. Whilst the ubiquitin-proteasome pathway may be important as a dominant mechanism for increased muscle protein degradation in some fast-developing murine models of cancer cachexia^32^, accelerated autophagy may be important for the more slow-developing cachexia observed in patients^10^.

To firmly conclude that autophagy is accelerated in muscle during cachexia, autophagy flux in target tissue (i.e. skeletal muscle) needs to be assessed. Very few studies address this issue, mainly because quantifying autophagic flux *in vivo* remains challenging. Accumulation of autophagic markers (such as SQSTM1 and MAP1LC3) could reflect either reduced autophagic clearance or induction of the process^33, 34^ and any estimates of autophagy flux without addressing both possibilities should be interpreted with caution. Penna *et al*.^11^ have tried to overcome these difficulties by assessing the level of autophagy markers in cachectic muscle tissue of animals following treatment with the microtubule destabilizing agent colchicine. They detect increased levels of MAP1LC3B-II, interpreted as increased autophagy flux^11^. Several other studies also indicate that increased autophagy is involved in cachexia. Upregulation of autophagy-related transcripts, such as MAP1LC3B, BNIP3 and GABARAPL1, has been observed in many different types of muscle atrophy^6^. In addition, denervation increases the muscular expression of MAP1LC3, GABARAPL1, ATG4b, VPS34, BNIP3, BNIP3L, Beclin1 and ATG12^35^, which are all involved in autophagy. Whether an elevated mRNA level of these genes reflects acceleration of the process in muscle remains undetermined.

Increased activity of lysosomal proteases is detected in cachectic animals, suggesting elevated autophagy flux^36^. Accordingly, the level of branched chain amino acids increases in serum from pancreatic cancer patients and mice with weight loss^37^. Remarkably, the raise in branched amino acids is detected before weight loss and cancer diagnosis^37^, indicating that accelerated autophagy occurs early in cachexia development. Furthermore, Sousa *et al*.^18^ recently reported that pancreatic cancer cells secrete compounds that are able to induce autophagy in stromal fibroblasts, which then release alanine to support survival of the cancer cells^18^. Moreover, increased protein level of MAP1LC3B-II and Beclin1 is detected in muscles of cachectic cancer patients^8^, suggesting modulation of the process also in humans. Our finding that there is an association between weight loss and autophagy-inducing bioactivity in blood samples from patients with GI and lung cancer is in line with an important role of autophagy in cachexia development.

Lack of association between weight loss and autophagy-accelerating bioactivity in the blood in the heterogeneous group of cancer patients included in the EPOS study, suggests that involvement of autophagy is partly dependent on cancer type and the prevalence of cachexia. However, the patients included in the EOPS study were mostly pre-treated and all were receiving opioid treatment. Opioids have been shown to modulate autophagy^38, 39^, and such compounds, if still present in the serum samples, may mask the activity of putative tumor-derived signaling compounds. Moreover, the samples from the EPOS study were from patients with many different types of cancer, such as breast and hematological cancers where cachexia is less frequent. Weight loss in these patients may therefore, to a larger extent, be caused by non-cachexia related factors, where autophagy possibly does not play the same role.

Strikingly, for the lung cancer patients, we found the association between autophagy-inducing bioactivity and weight loss to be gender-specific (in men only). Interestingly, a greater role of autophagy in cachectic males has been suggested before^40^. The authors found a greater increase in autophagy markers in the heart of male cachectic mice than in female. They propose that this contributes to greater prevalence of cardiac atrophy in male vs. female mice. Furthermore, loss of grip strength, as well as loss of total body weight is more pronounced in male cancer patients^22, 41^. However, more studies with larger groups are needed to address issues of gender-specific involvement of autophagy in cachexia in detail. Moreover, cachexia is characterized by loss of lean body mass and is not necessarily reflected in loss of total body weight^2, 42^. Whether autophagy acceleration correlates better with specific loss of lean tissue in humans requires further studies, preferably including information on fat/muscle composition^42^. Nevertheless, we note that men generally have a higher muscle/fat ratio and since the association between weight loss and autophagy-inducing activity in lung cancer patients were male-specific, this suggests a stronger role of autophagy in muscle loss.

For autophagy to contribute to cachexia it must be accelerated systemically in lean tissue. Studies in mice have shown that autophagy can be accelerated in various tissues in the body as a starvation response, and genetically modified mice defective in autophagy survive only 12–24 h before they die of undernourishment^14, 15^. This demonstrates that systemic induction of autophagy is a crucial physiological process during starvation, but how this response is coordinated is currently not clear. In adult mice, induced autophagy seems to occur without clear changes in the levels of circulating amino acids^15^. We speculate that in some cachectic cancer patients, tumor-secreted substances mimic normal starvation responses. Micro-environmental crosstalk where cancer cells fuel their own growth by stimulating autophagy in stroma cells has previously been proposed^17, 18^, and suggested to translate to a systemic principle in cachexia^17^. However, the identity of the tumor-derived signaling compound that drives autophagy in stroma is unknown.

Cancer cachexia is tumor driven and treatments that efficiently target the tumor also influence cachexia^43^. Mice lacking the *IL-6* gene develop mature onset obesity and the cytokine is needed to mobilize energy during prolonged exercise^44, 45^. Thus, IL-6 has an established role as a central regulator of metabolism in normal physiology. IL-6 has previously been shown to increase the turnover of long lived proteins as well as increasing the activity of lysosomal proteases^46^. Furthermore, IL-6 levels are frequently found elevated in cancer patients with an association to weight loss and shorter survival^47–49^. Also, the cytokine is sufficient to cause weight loss in mice^50–52^. In mice harboring C26 or Yumoto cell tumors, muscle wasting can be blocked by IL-6 blocking agents^53–55^ and administration of the IL-6 receptor neutralizing antibody tocilizumab has shown promising results in attenuating muscle loss in humans^56, 57^. Equally, the MEK inhibitor selumetinib inhibits production of IL-6 and reverses skeletal muscle wasting in patients with advanced cholangiocarcinoma^58^. We found that tocilizumab as well as knock down of IL-6 expression efficiently blocked cancer cell-induced autophagy acceleration, consistent with an important role for IL-6 in autophagy regulation.

IL-6 can initiate intracellular responses by two main modes of action. Classical signaling (cis-signaling) occurs when IL-6 binds to transmembrane IL-6 receptor on the target cell and the complex associates with transmembrane co-receptor gp130. Alternatively, IL-6 can complex with soluble IL-6 receptor and thereafter locate to gp130 on cellular membranes (trans-signaling)^59^. We show that IL-6 alone can accelerate autophagy, but that this is dependent on IL-6 receptor expression. In cells with a low expression of the receptor (e.g. myocytes) we were unable to detect any consistent induction of the process in response to IL-6. Rather, we show that trans-signaling is a very potent autophagy-accelerating mechanism, also in muscle cells. Interestingly, mice with a transgenic expression of IL-6/IL-6R appear smaller than single transgenic mice that express only IL-6 or IL-6 receptor^60^, suggesting that IL-6 trans-signaling is more important than cis-signaling for regulation of body weight. IL-6 receptors are primarily expressed on hepatocytes and some lymphoid cells^25^. IL-6 trans-signaling may therefore be especially important in tissues that are severely affected during cachexia, such as lean tissue. Accordingly, we found that IL-6 trans-signaling accelerates autophagy in differentiated muscle cells (C2C12 myotubes), whereas IL-6 (cis-signaling) did not. Interestingly, neither mode of IL-6 signaling accelerated autophagy in C2C12 myoblasts. This is despite a strong ability of hyper-IL-6, an IL-6/IL-6 receptor fusion construct, to induce STAT3 phosphorylation. This suggests that STAT3 phosphorylation is not sufficient to cause autophagy acceleration. Others have shown that IL-6 elevates the activity of AMPK^61^, a central regulator of cellular energy balance and inducer of autophagy. Whether IL-6 can regulate autophagy by this mode of action and whether such a regulation is dependent on cell type or state of differentiation is yet to be determined. Furthermore, whereas this study highlights the ability of IL-6 trans-signaling to induce autophagy, the role of IL-6 trans-signaling in affecting other potential mechanisms to muscle and weight loss (such as increased proteasomal activity) still remains to be investigated. It also remains to be determined whether trans-signaling-induced regulation of these processes depends on differentiation status of muscle cells.

IL-6 trans-signaling is counteracted by soluble gp130 and can only occur if the level of soluble IL-6R increases beyond the antagonistic activity of soluble gp130^59^. Interestingly, the level of IL-6R is found to be increased in both lung lysates and sera from patients with lung adenocarcinoma^29^, suggesting a systemic effect of IL-6 trans-signaling in these patients. It is, however, important to acknowledge that increased bioactivity of IL-6/IL-6 receptor complexes *in vivo* is not necessarily dependent on an increase in the level of soluble IL-6 receptor. The bioactivity of IL-6/IL-6 receptor complexes could potentially also rise if the soluble gp130 present in circulation is reduced or restricted from antagonizing IL-6 trans-signaling. Whether this occurs in cachectic patients is yet to be determined.

Interestingly, Miller *et al*.^62^ recently demonstrated in an *in vivo* model of cancer cachexia that IL-6 trans-signaling is important for weight loss, early mortality and reduced muscle and adipose tissue mass, consistent with cachexia^62^. Furthermore, in another recent study by Katheder *et al*.^63^, it was shown that Ras-driven tumor development in Drosophila is supported by local and systemic autophagy in an IL-6 dependent manner^63^. Our results, together with these recent *in vivo* studies could suggest that development of cancer cachexia might be prevented by compounds that either restrict IL-6 trans-signaling or interfere with autophagy. Tocilizumab has been reported to reverse cachexia in some cancer patients^56, 57^. This is also the case for the MEK inhibitor selumetinib, which is known to inhibit IL-6 production^58^. Similarly, the benefits of n-3 fatty acids in preventing loss of lean body mass in lung cancer patients undergoing chemotherapy^64^ may relate to their ability to suppress IL-6 production^65^. We show that IL-6 trans-signaling is a potent driver of autophagy in muscle cells, indicating that cachectic patients may benefit from treatment that target IL-6 trans-signaling specifically. Targeting of IL-6 trans-signaling may also allow the beneficial anti-inflammatory actions of IL-6 cis-signaling to occur and reduce the infection hazard associated with the blockade of IL-6 cis-signaling^66^. Soluble gp130Fc, that inhibits IL-6 trans-signaling specifically, has already undergone phase I clinical trials^66^. However, further trials to validate the benefit of this approach to prevent or reverse cachexia are strongly encouraged. Targeting autophagy in cachexia treatment might also be beneficial and compounds that inhibit autophagy (chloroquine or hydroxychloroquine) have been in clinical use against various conditions including malaria and rheumatoid arthritis. When used against these disorders, chloroquine has mostly shown minor side effects^67^. However, long term use of chloroquine has been associated with retinal toxicity and the development of age-related macular degeneration (AMD)^68–70^ and its potential benefit in cachexia treatment should be weighed against the risk of developing such disorders. Regardless, compounds that slow, halt or reverse the development of cancer cachexia have the potential to increase life expectancy and/or the quality of life for a large number of cancer patients.

## Materials and Methods

### Patient material

75 serum samples from patients with various cancer types (Supplementary Table S4) were obtained from the Human Pharmacogenetic Opioid Study (EPOS), St.Gallen, Switzerland; 128 heparin plasma samples from oesophageal, gastric or pancreatic cancer patients from the Edinburgh Cancer Cachexia Cohort (ECCC) Study, Edinburgh, Scotland; 84 serum samples from the Central Norway Lung Cancer Biobank (CNLCB), Trondheim, Norway; and 148 serum samples from healthy controls from the Nord-Trøndelag Health Survey (HUNT), Trondheim, Norway. Healthy controls were matched to lung cancer patients based on gender, age and smoking habit (smoking vs. non-smoking). Autophagy flux was evaluated in all samples. 5 patients from CNLCB and 1 patient from ECCC were omitted due to indications of treatment interfering with the autophagy assay (doxycycline analogs). 4 more sera from CNLCB were omitted from analyses that included weight loss, as this information was lacking. 23 randomly selected autophagy-inducing sera and 23 non-autophagy inducing sera from gender/smoking-habit/age matched patients (CNLCB) were used for screening of IL-6 levels. Weight loss (reported by the patient) is detected as % loss during 3 (CNLCB) or 6 (ECCC and EPOS) months prior to sampling (Supplementary Table S5). Experiments were performed in accordance with the approval from the local ethical committee (EPOS^71^: The Regional Committees for Medical and Health Research Ethics (REC), Norway, OPI 03–006/sks, CNLCB/controls: The Regional Committees for Medical and Health Research Ethics (REC), Norway, 4.2005.777, ECCC: Lothian Regional Ethics Committee (REC), Scotland, 06/S1103/75). Written informed consent was received from participants prior to inclusion in the study and the study was performed in conformity with the declaration of Helsinki.

### Animal models

TOV21G (5 × 10^6^ cells) were inoculated subcutaneously into the flank region of female BALB/c nu/nu mice (Taconic, Denmark) and body weight was monitored regularly up to 25 days post-inoculation. G-361 (8 × 10^6^ cells), A2058 or MKN-1 (2 × 10^6^ cells) were inoculated subcutaneously into the flank region of female Hsd:Athymic Nude-*Foxn1* ^*nu*^ mice (Harlan Laboratories, Netherlands) and body weight was monitored regularly for 27–31 days post-inoculation. Experiments were approved by the National Animal Research Authorities and carried out according to the European Convention for the Protection of Vertebrates used for Scientific Purposes (TOV21G: FOTS ID 3376. Experiments using G-361, A2058 and MKN-1 were performed according to the regulations effective in the Canton of Basel-City, Switzerland, license number BS-2186).

### Cell culturing and reagents

Cell culture conditions and reagents used are specified in supplemental materials and methods.

### Co-culture experiments and conditioned media

In co-culture experiments, seeded number of autophagy reporter cells (15000 cells per well) was constant in all cell-cell combinations and the number of the respective cell lines was adjusted so that 80–90% confluence was reached at the time of autophagy assessment. During co-culture experiments where the cell lines were separated, the cell lines were separated using well inserts with a semipermeable membrane (pore size 4 µm). Autophagy flux was assessed following 3 days co-culturing/semi co-culturing when cells reached approximately 80% confluence. Cell lines were in these experiments held in DMEM supplemented with FBS (10%) and gentamicin (0.05 mg/ml) in 24-well plates.

Conditioned medium (CM) was made by 3 days culturing of cells (unless otherwise indicated). For TOV21G, full growth medium (15% FBS) was used and the cells were 80% confluent after 3 days. For the remaining cell lines, the cells were 80% confluent when changing from full medium to serum free (SF) medium (see supplemental experimental procedures for media components) and the cells were left for 3 days before collecting the medium. Autophagy-inducing effect of TOV21G conditioned medium (CM) was compared to TOV21G growth medium incubated for 3 days under the same conditions as CM, but without cells. When monitoring the effect of CM from SF conditions, CM was diluted 1:3 in DMEM (Sigma D5796), FBS was added (10% final concentration) and autophagy-inducing effect was compared to fresh medium (10% FBS). Relative effects of CM on autophagy in reporter cells varied between seedings and CM batch. However, data presented in each figure panel is from the same experiment and bars are directly comparable.

### Immunoblotting

HEK293 autophagy reporter cells were treated with Bafilomycin A1 (100 nM) for 3 or 6 hours at the end of 3 days incubation with/without TOV21G CM and then scraped on ice in lysis buffer. C2C12 myocytes were treated with Bafilomycin A1 (100 nM) for 3, 6 or 24 hours at the end of 3 days incubation with/without rIL-6 (20ng/ml) or CM from hyper IL-6-producing CHO cells (0.1%) before they were scraped on ice in lysis buffer. HEK293 autophagy reporter cells and U2OS cells treated with Bafilomycin A1 (100 nM) for 1, 6 or 20 hours with/without rIL-6 (20 ng/ml) were trypsinized, washed twice in PBS and lysed (reporter cells) or scraped on ice in lysis buffer (U2OS cells). All cell cultures were approximately 80% confluent at the time of lysis. Bafilomycin A1 (a vacuolar type H^+^-ATPase [V-ATPase] inhibitor) was used to disrupt lysosomal function and fusion between autophagosomes and lysosomes. Blockage of autophagy in this manner causes an accumulation of proteins like MAP1LC3B and SQSTM1 that are selectively degraded in the lysosomes via autophagy. Compounds or conditions that rise the autophagy flux will cause an additional increase in the level of MAP1LC3B and SQSTM1 when combined with the lysosomal inhibitor compared to cells treated with the inhibitor alone. For studying bioactivity of hyper IL-6, IL-6/IL-6R, IL-6, IL-8 and VEGF, cells were cultivated in medium containing 0.1% FBS for 2 hours before treatment and protein isolation by cell scraping on ice. All cells were lysed in a buffer containing 8 M Urea, 0.5% (v/v) Triton X-100, 100 mM DTT, 1xComplete^®^ protease inhibitor (PI), and 8% phosphatase inhibitor cocktail I and III, respectively (Sigma). Protein concentration was determined by BioRad protein assay (BioRad). Equal amounts of proteins were separated using NuPAGE^®^ Novex^®^ 12% or 4–12% Bis-Tris Gels (Invitrogen). Membranes were blocked and antibodies diluted in a 1:1 mixture of Odyssey blocking buffer (Li-Cor) and TBST (20 mM Tris, pH 7.6, 137 mM NaCl with 0.1% Tween 20). Bound antibodies were imaged by near-infrared fluorescence using fluorescent dye-labeled secondary antibodies and Odyssey NIR scanner (Li-Cor Biosciences). Images were processed using the Li-Cor Odyssey software image studio 3.1. Antibodies used for immunostaining are listed in Supplemental materials and methods. All immunoblots displayed in main figures are cropped. Images of the uncropped immunoblots are displayed in Supplementary Figure S4.

### Autophagy reporter system

Autophagy flux was quantified using flow cytometry measuring mean green fluorescence intensity per cell of live HEK293 cells expressing the GFP-SQSTM1 fusion protein as previously described^19^. Briefly, the expression of the fusion gene was induced by doxycycline. Since the SQSTM1 protein is selectively degraded by autophagy, the degradation of the fusion protein can be measured as loss in GFP monitored by flow cytometry. The autophagy mediated degradation of this fusion protein occurs at a basal rate and can be increased by different stimuli. The green fluorescent intensity after a certain time point in untreated (vehicle treated) cells is compared to the intensity in treated cells. Increased loss of GFP-SQSTM1 following treatment is interpreted as increased autophagy flux and displayed in figures as relative autophagy flux. HEK293 autophagy reporter cells were seeded in 24-well plates, incubated for 1 day and treated as indicated for 3 days (unless otherwise indicated) in the presence of doxycycline (1 ng/ml) before assessment of fusion protein degradation by flow cytometry. Protein degradation was determined as the loss of green fluorescent signal compared to control. Triplicate wells (unless otherwise stated) were used and signal measured in 10,000 cells/well. For experiments using patient material or co-cultures/semi-co-cultures, reporter cells were seeded in 24- or 48-well plates in the presence of doxycyclin (1 ng/ml) and incubated for 48 hours before promotor shut-off (doxycyclin removal/medium exchange). The cells were washed in PBS and added DMEM medium supplemented with human serum or plasma (10%) or FBS (10%). Following 16 hours incubation, the degradation rate of the fusion protein was determined. Cells were seeded to reach about 80% confluency at the end of the experiment. All samples were calculated relative to the internal standard, FBS, included in all experiments. Healthy control and patient sera (CNLCB) were tested using duplicate wells and data represent mean of at least two independent experiments. Plasma from GI cancer patients (ECCC) was tested using single wells. To ensure result reliability, randomly selected samples (n = 18) were tested again using duplicate wells. In these cases, data represent mean of both experiments. Sera from patients included in EPOS were tested in duplicate wells and data represent mean. In experiments using combinations of patient sera and tocilizumab or sgp130Fc, HEK293 autophagy reporter cells were seeded in 48-well plates, incubated for 1 day and treated for 3 days in the presence of doxycycline (1 ng/ml) before assessment of fusion protein degradation by flow cytometry.

### Fiber cross section analysis

Frozen cross-sections (8 µm) were prepared from OCT-embedded tibialis muscle using a CryoStar NX70 (Thermo Scientific) and co-stained for laminin (Sigma-Aldrich, L9393) and myosin heavy chain type IIa or IIb to distinguish each fiber type (SC-71 for type IIa and BF-F3 for type IIb, obtained from Developmental Studies Hybridoma Bank at the University of Iowa), followed by staining with secondary antibodies Alexa 488 anti-rabbit (Invitrogen, A-11070) for laminin, Alexa 350 anti-mouse (Invitrogen, A-21120) for type IIa, and Alexa 555 anti-mouse (Invitrogen, A-21426) for type IIb. Images of the entire tibialis muscle section were acquired using a VS120 slide scanner (Olympus Corporation, Tokyo, Japan) and processed into tiles to enable imaging analysis of the entire section for each muscle. Distribution of myosin heavy chain fiber types and the cross section area of individual fibers (ranging from several hundreds to a few thousands) in the scanned section were analyzed using the proprietary image analysis platform ASTORIA (Automated Stored Image Analysis) based on Matrox MIL9 technology developed by Novartis/Preclinical Safety.

### Multiplex analysis

Cytokine, chemokine and growth factor levels in TOV21G CM were determined using a microsphere-based Bio-Plex Human Cytokine 27-plex assay (Bio-Rad, Hercules, CA), according to the manufacturer’s instructions. Levels of IL-6, IL-8 and VEGF were measured using a Bio-Plex Pro Assay (BioRad) (n = 2 for 1 day and n = 3 for 3 days CMs).

### CRISPR/Cas9-mediated IL-6 knock down

Guide RNAs targeting the IL-6 gene were selected using the CRISPR Design tool from MIT and the Zhang lab (http://crispr.mit.edu/). A guide pair targeting exon 2 (primer A: 5′-GTGTGGGGCGGCTACATCTT[TGG]-3′ and primer B: 5′-AACGAATTGACAAACAAATT[CGG]-3′) and a guide pair targeting exon 3 (primer A: 5′-CAGCCATCTTTGGAAGGTTC[AGG]-3′ and primer B: 5′-AAAGATGGATGCTTCCAATC[TGG]-3′) and their complementary sequences were given 5′ overhangs to fit into the BbsI cloning site of the vector, annealed and cloned into the pSpCas9n(BB)-2A-GFP (PX461) plasmid (a gift from Feng Zhang (Addgene plasmid #48140)^72^). Verification of correct sequence insertion was done using restriction enzyme cutting followed by sequencing of selected clones using the LKO.1 5′ human U6 promoter (5′-GACTATCATATGCTTACCGT-3′ (Weinberg Lab)). TOV21G cells were seeded in 10 cm culture plates, incubated 1 day (about 80% confluence) and transfected with PX461 without (6 µg plasmid/ml medium) or with inserted guide sequence pairs (3 µg of each guide plasmid/ml medium) using X-tremeGENE HP DNA transfection reagent (Roche) with a ratio of transfection reagent (µl) to plasmid (µg) of 3:1. Following 1 day incubation the transfection medium was exchanged with fresh growth medium. After 2 additional days the cells were trypsinized, washed twice and resuspended in PBS. Cells with PX461 uptake (GFP expression) was sorted (using BD FACSAria™ Fusion) and seeded 1cell/well in 96-well plates. IL-6 was not completely knocked out in any of the formed clones. However, clones with the greatest IL-6 protein reduction were used in experiments.

### IL-6 ELISA

IL-6 level in TOV21G CM were determined using a Human IL-6 ELISA Set (BD Biosciences, cat. no. 555220) according to the manufacturers protocol.

### IL-6 bioassay

Bioactivity of IL-6 in CM from TOV21G cells were tested using an established bioassay based on the IL-6-dependent survival of B9 cells^73^. See supplemental materials and methods.

### Statistics

SPSS, Microsoft Excel, or GraphPad Prism (GraphPad Software, Inc., La Jolla, CA) were used for statistical work. The tests performed are specified when individual results are presented. For cell line-based results a normal distribution was assumed. p-values < 0.05 were considered statistically significant and when labeled with * (or **, or ***), this indicates the degree of statistical significance. To test the potential difference in continuous variables such as weight loss, the t-test for independent groups was used, and to test for differences in percentages between groups, the Pearson chi square test was used. To evaluate whether a gradual increase in autophagy was associated with a gradual weight reduction, we used a scatter plot and applied linear regression with autophagy flux as the independent variable, and percent reduction in weight as the continuous outcome variable. The regression coefficient then denoted the percentage change in weight loss by one unit change in autophagy. By using 50 percent change in autophagy as the unit in the regression analysis, we were able to estimate the expected change in weight by 50% change in autophagy. The same analysis was applied when looking at the association between IL-6 level and CRP or weight reduction, but then with IL-6 as the independent variable and CRP or percent weight loss, respectively, as the continuous outcome variable. To evaluate the precision, the estimate was accompanied by the 95% confidence interval and a p -value.

### Data presentation

Figures were mounted using Canvas 14 (ACD systems).

## Electronic supplementary material

Supplemental material
