# Supplementary material for: Cancer cachexia associates with a systemic autophagy-inducing activity mimicked by cancer cell-derived IL-6 trans-signaling
Source: Sci Rep. 2017 May 17;7:2046. doi: 10.1038/s41598-017-02088-2 (PMC5435723; doi:10.1038/s41598-017-02088-2)
Supplement: Supplementary file 1 — Supplemental material [file 41598_2017_2088_MOESM1_ESM.pdf]

Supplementary materials

Cancer cachexia associates with a systemic autophagy-inducing activity mimicked by cancer cell-derived IL-6 trans-signaling

Kristine Pettersen, Sonja Andersen, Simone Degen, Valentina Tadini, Joël Grosjean, Shinji Hatakeyama, Almaz N. Tesfahun, Siver Moestue, Jana Kim, Unni Nonstad, Pål R. Romundstad, Frank Skorpen, Sveinung Sørhaug, Tore Amundsen, Bjørn H. Grønberg, Florian Strasser, Nathan Stephens, Dag Hoem, Anders Molven, Stein Kaasa, Kenneth Fearon, Carsten Jacobi, Geir Bjørkøy

SUPPLEMENTAL MATERIALS AND METHODS

Cell culture conditions and reagents

Cells were maintained in a humidified atmosphere of 5% CO_2; 95% air at 37°C. TOV21G cells from ATCC (CRL-11730) were cultured in 42.5% Medium 199+GlutamaxTMI (Gibco 41150) and 42.5% medium MCDB 105 (Sigma M6395, dissolved in dH_2O to 1L final volume, added NaHCO3 (1.5 g/L) and adjusted to pH 7.3), supplemented with Fetal Bovine Serum (FBS, 15%,) and gentamicin (0.05 mg/ml). HEK293 GFP, GFP-p62 and GFP-p62^{L341A} flip-in cells (a kind gift from Prof. T. Johansen) and HeLaS3 cells (a kind gift from Prof. Marit Otterlei) were cultured in DMEM (Sigma D5796) supplemented with FBS (10%) and gentamicin (0.05 mg/ml). SW620 cells from ATCC (CCL-227) were cultured in Leibovitz's L-15 medium (BioWhittaker 12-700-F), supplemented with L-glutamine (2 mM), FBS (10%) and gentamicin (0.05 mg/ml). Caco-2 and A427 cells from ATCC (HTB-37 and HBT-53, respectively) were cultured in Eagle medium (Sigma, M5650) supplemented with L-glutamine (2 mM), Sodium pyruvate (NaP; 1%), gentamicin (0.05 mg/ml), and FBS (20% and 10%, respectively). A549 cells from ATCC (CCL-185) were cultured in DMEM/F-12 medium (Sigma D8437) supplemented with FBS (10%) and gentamicin (0.05 mg/ml). A-172 from ATCC (CRL-1620) were cultured in DMEM (Sigma D6429) supplemented with NaP (1%), gentamicin (0.05 mg/ml), and FBS (10%). U2OS cells from ATCC (HTB-96) were cultured in RPMI 1640 (Sigma R8758) supplemented with L-glutamine (0.6 mM), gentamicin (0.02 mg/ml), and FBS (10%). A2058 from ATCC (CRL-11147) were cultured in DMEM and Glutamax media (Invitrogen, 31966) supplemented with FBS (10%), NaHCO3 (1.5 g/l, Invitrogen) and Pen/Strep/Fungizone (PSF, 1%; PAA#P11-002). MeWo cells from ATCC (HTB-65) were cultured in MEM (Invitrogen, 31095029) supplemented with FBS (10%), non-essential amino acids (NEAS, 1x, Gibco #11140), NaP (1 mM), NaHCO3

(1.5 g/l, Invitrogen) and PSF (1%). G361 cells from ATCC (CRL-1424) were cultured in McCoy's medium (Invitrogen, 26600) supplemented with FBS (10%) and PSF (1%). MKN-1 cells from RIKEN (RCB1003) were cultured in RPMI-1640 (Sigma, R7638) supplemented with FBS (10%) and PSF (1%). B9 cells (a kind gift from Drs. L. A. Aarden and M. Helle) were cultured in RPMI (Sigma R8758) supplemented with FBS (heat inactivated, 10%) and IL-6 (about 1 ng/ml) isolated from peripheral blood leukocytes (PBL) according to the following procedure: PBL are washed twice in Hanks Balanced Salt Solution (HBSS) and resuspended in RPMI (Sigma R8758) supplemented with FBS (heat inactivated, 5%) and LPS (0.1 μg/ml). Cells are incubated for 24 hours before the supernatant is harvested and frozen at -20°C. IL-6 is tested against previous harvested IL-6. Exact IL-6 concentration will vary from batch to batch. C2C12 myocytes from ATCC (CRL-1772) were cultured in DMEM (Sigma D5769), supplemented with NaP (0.11g/L), FBS (10%,) and gentamicin (0.05 mg/ml). When differentiating C2C12 cell into myotubes, myoblasts were seeded in conventional medium. Once the culture reached 80-90% confluency (two days) medium was exchanged to (Sigma D5769), supplemented with NaP (0.11g/L), Horse Serum (2%,) and gentamicin (0.05 mg/ml). Following five days, myotubes had formed and experiments were initiated.

Serum-free media used to make conditioned media were as follows: A-2058: phenol-red free DMEM (Invitrogen #11880), PSF (1%) and NaHCO3 (1.5 g/L), MeWo: phenol-red free MEM (Invitrogen #51200046), NEAS (1x), NaP (1 mM) and NaHCO3 (1.5 g/L), G-361: as full medium without FCS, MKN-1: phenol-red free RPMI (Sigma R7509) with PSF (1%).

rIL-6 (Invitrogen, cat.no. PHC0066), rIL-8 (Gibco, cat.no. PHC0084), rVEGF (R&D systems, cat.no. 293-VE), Tocilizumab/RoACTEMRA (a humanized monoclonal antibody against the interleukin-6 receptor, Roche) IL-6-specific ab (R&D systems, Cat.no. AF-206-NA), Hanks

balanced salt solution (HBSS, Gibco), recombinant IL-6 receptor alpha (R&D systems, cat.no. 227-SR-025), Recombinant Human gp130 Fc Chimera Protein, CF (spg130Fc, R&D systems, cat.no. 671-GP-100 or a kind gift from Prof. J. Scheller [1]), and conditioned medium from hyper IL-6-producing Chinese hamster ovary (CHO) cells (a kind gift from Prof. J. Scheller [2]) were used as specified.

IL-6 bioassay

B9 cells were seeded in 96-well plates (5000 cells/well) in RPMI (Sigma R8758) supplemented with FBS (heat inactivated, 5%) and CM from TOV21G cells (1:2) or a series of rIL-6 concentrations ranging from 0-64 pg/ml (Standard curve). Following 3 days, 5 mg/ml MTT (3-(4,5-dimethylthiazol-2-yl)-2,5-diphenyltetrazolium bromide) in PBS (20 µl/well) was added and the culture incubated for 4 hours at 37°C. Thereafter, 150 µl of the supernatant was removed and replaced with 100 µl 0.04 M HCl in isopropanol. The plate was shaken until formazan dissolved and optical density was measured using a microplate reader at 570 nm.

Antibodies

Anti-SQSTM1 (Progen, Cat.no. GP62-C), anti-LC3B (D11, Cell Signaling Tech., Cat.no. 3868), anti-β-actin/ACTB and anti-β-tubulin/TUBB (Abcam, cat.no. ab6276 and ab6046, respectively), anti-phosphoAKT^{Ser473} (Cell signaling, (D9E) XP, cat.no. 4060), anti-phosphoSTAT3^{Tyr705} and anti-MAPK (Erk1/2) (Cell signaling, cat.no. 9145S and 9107 respectively), and anti-PCNA (Santa cruz biotechnology (PC10), cat.no. sc-56).

SUPPLEMENTAL FIGURES AND TABLES

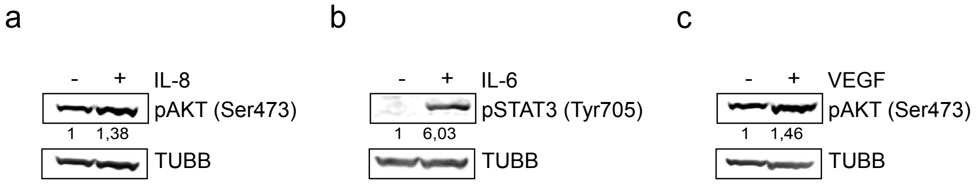

Figure S1. Bioactivity of recombinant IL-8, IL-6 and VEGF. A-C) Protein levels of pAKTSer473 and pSTAT3Tyr705 in HEK293 reporter cells treated for 5 minutes with rIL-8 (10 ng/ml), rIL-6 (20 ng/ml) and VEGF (2 ng/ml). Signal intensity of the phospho-protein band were normalized against β-tubulin/TUBB as indicated.

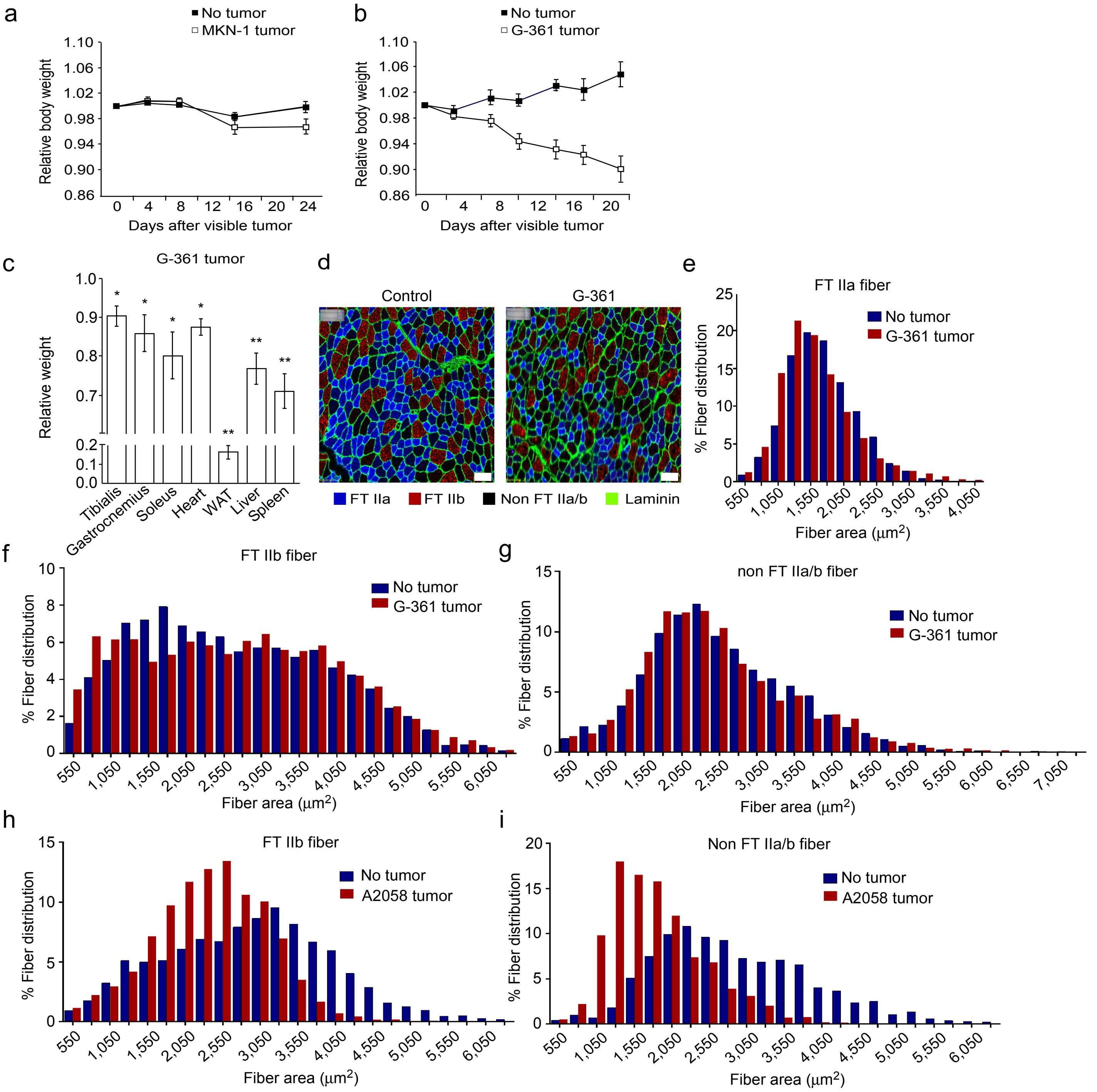

Figure S2. Cachectic effects of autophagy-inducing and non-inducing cell lines A-B) Mean relative body weight (at indicated time intervals) ±SEM of MKN-1 (n=10) and G-361 (n=12) tumor-bearing mice and control mice (n=8), respectively. C) Mean relative muscle, white adipose tissue (WAT) and organ weight ±SEM of G-361 (n=12) tumor-bearing mice in comparison to control mice (n=8). *p<0.05, **p<0.01 versus control (Student t-test). D) Cross section of tibialis muscle from control and G-361 tumor-bearing mice. Fast Twitch (FT) IIa, IIb muscle fibers and laminin are immunostained as indicated. Scale bar=100μm. E-G) Mean tibialis muscle Fast Twitch (FT) IIa, b and non FT fiber area in controls (n=5) and G-361 tumor-bearing mice (n=5). H-I) Mean tibialis muscle FT IIb and non FT fiber area in controls (n=3) and A2058 tumor-bearing mice (n=4).

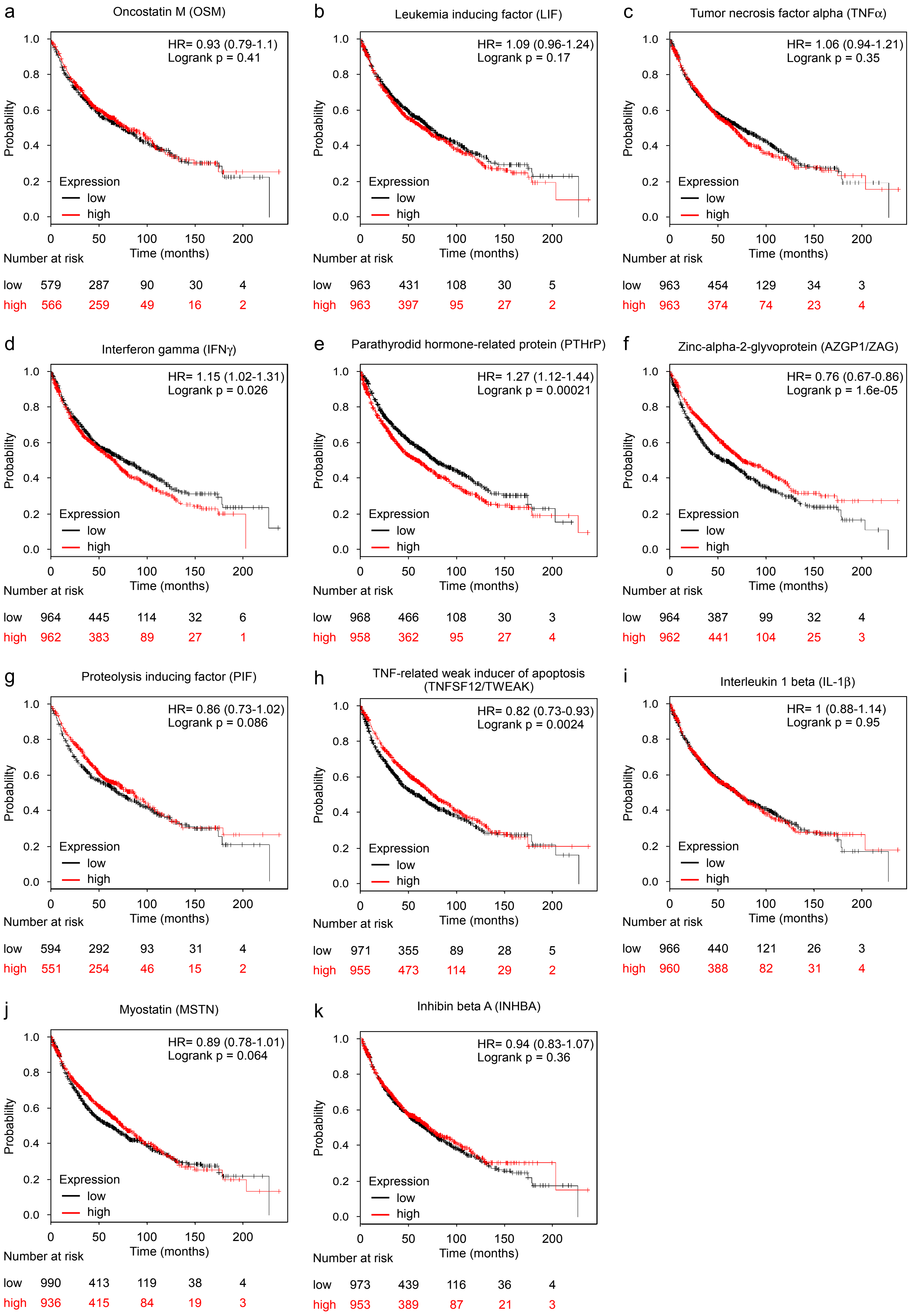
a
Oncostatin M (OSM)
HR= 0.93 (0.79-1.1)
Logrank p = 0.41
Expression
low
high
Probability
Time (months)
Number at risk
low 579 287 90 30 4
high 566 259 49 16 2
b
Leukemia inducing factor (LIF)
HR= 1.09 (0.96-1.24)
Logrank p = 0.17
low 963 431 108 30 5
high 963 397 95 27 2
c
Tumor necrosis factor alpha (TNFα)
HR= 1.06 (0.94-1.21)
Logrank p = 0.35
low 963 454 129 34 3
high 963 374 74 23 4
d
Interferon gamma (IFNγ)
HR= 1.15 (1.02-1.31)
Logrank p = 0.026
low 964 445 114 32 6
high 962 383 89 27 1
e
Parathyrodid hormone-related protein (PTHrP)
HR= 1.27 (1.12-1.44)
Logrank p = 0.00021
low 968 466 108 30 3
high 958 362 95 27 4
f
Zinc-alpha-2-glyvoprotein (AZGP1/ZAG)
HR= 0.76 (0.67-0.86)
Logrank p = 1.6e-05
low 964 387 99 32 4
high 962 441 104 25 3
g
Proteolysis inducing factor (PIF)
HR= 0.86 (0.73-1.02)
Logrank p = 0.086
low 594 292 93 31 4
high 551 254 46 15 2
h
TNF-related weak inducer of apoptosis (TNFSF12/TWEAK)
HR= 0.82 (0.73-0.93)
Logrank p = 0.0024
low 971 355 89 28 5
high 955 473 114 29 2
i
Interleukin 1 beta (IL-1β)
HR= 1 (0.88-1.14)
Logrank p = 0.95
low 966 440 121 26 3
high 960 388 82 31 4
j
Myostatin (MSTN)
HR= 0.89 (0.78-1.01)
Logrank p = 0.064
low 990 413 119 38 4
high 936 415 84 19 3
k
Inhibin beta A (INHBA)
HR= 0.94 (0.83-1.07)
Logrank p = 0.36
low 973 439 116 36 4
high 953 389 87 21 3

Figure S3. Lack of clear prognostic value of expression level for proposed cachexia inducing signaling substances and survival of lung cancer patients. A-K) Tumor expression of the indicated genes (split by mean mRNA level) predicts increased or decreased survival of lung cancer patients as indicated. HR=hazard ratio. The data are obtained using the kmplot.com database tool [3,4]

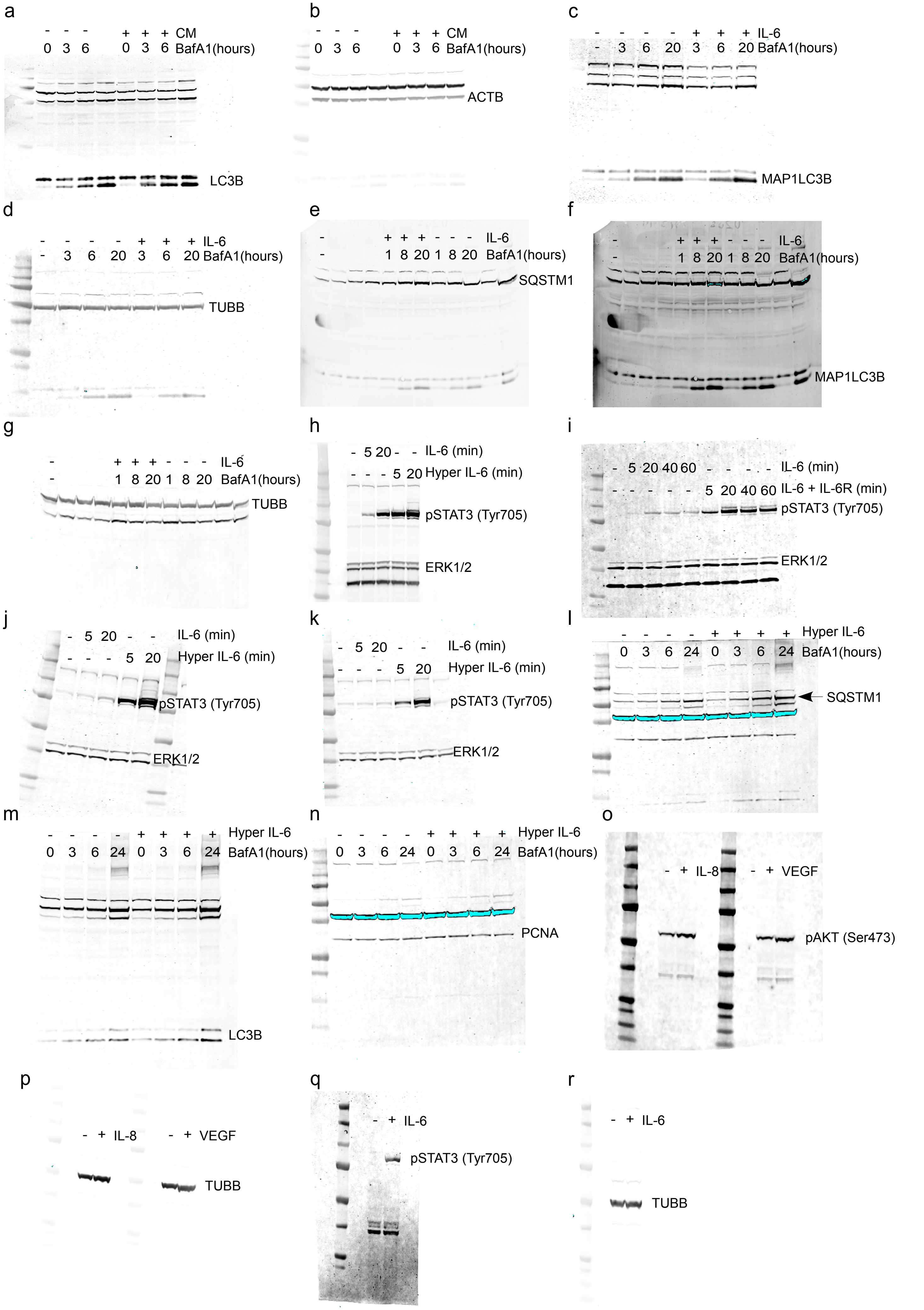
a
- - - + + + CM
0 3 6 0 3 6 BafA1(hours)
LC3B
b
- - - + + + CM
0 3 6 0 3 6 BafA1(hours)
ACTB
c
- - - - + + + IL-6
- 3 6 20 3 6 20 BafA1(hours)
MAP1LC3B
d
- - - - + + + IL-6
- 3 6 20 3 6 20 BafA1(hours)
TUBB
e
- + + + - - - IL-6
- 1 8 20 1 8 20 BafA1(hours)
SQSTM1
f
- + + + - - - IL-6
- 1 8 20 1 8 20 BafA1(hours)
MAP1LC3B
g
- + + + - - - IL-6
- 1 8 20 1 8 20 BafA1(hours)
TUBB
h
- 5 20 - - IL-6 (min)
- - - 5 20 Hyper IL-6 (min)
pSTAT3 (Tyr705)
ERK1/2
i
- 5 20 40 60 - - - - - IL-6 (min)
- - - - - 5 20 40 60 IL-6 + IL-6R (min)
pSTAT3 (Tyr705)
ERK1/2
j
- 5 20 - - IL-6 (min)
- - - 5 20 Hyper IL-6 (min)
pSTAT3 (Tyr705)
ERK1/2
k
- 5 20 - - IL-6 (min)
- - - 5 20 Hyper IL-6 (min)
pSTAT3 (Tyr705)
ERK1/2
l
- - - - + + + + Hyper IL-6
0 3 6 24 0 3 6 24 BafA1(hours)
SQSTM1
m
- - - - + + + + Hyper IL-6
0 3 6 24 0 3 6 24 BafA1(hours)
LC3B
n
- - - - + + + + Hyper IL-6
0 3 6 24 0 3 6 24 BafA1(hours)
PCNA
o
- + IL-8
- + VEGF
pAKT (Ser473)
p
- + IL-8
- + VEGF
TUBB
q
- + IL-6
pSTAT3 (Tyr705)
r
- + IL-6
TUBB

Figure S4. Uncropped version of immunoblots displayed in main figures and figure S1. Uncropped version of immunoblot displayed in main figure 2F (A-B), 3D (C-D), 3E (E-G), 5A (H), 5C (I), 5E (J), 5F (K), 5G (L-N), figure S1A and C (O-P) and figure S1B (Q-R).

Table S1. Cytokine, chemokine and growth factor levels (pg/ml) in conditioned medium from TOV21G cells following 1 and 3 days of cultivation. OOR= Out of range.

Factor	Control	1 day	3 days
IL-1beta	OOR <	OOR <	0.61
IL-1ra	3.70	15.10	62.49
IL-2	OOR <	0.63	3.12
IL-4	OOR <	0.26	0.88
IL-5	OOR <	OOR <	1.14
IL-6	OOR <	1475.64	7158.93
IL-7	OOR <	2.08	7.42
IL-8	OOR <	509.21	1909.53
IL-9	OOR <	2.08	9.54
IL-10	OOR <	2.78	10.65
IL-12 (p70)	OOR <	13.24	80.65
IL-13	OOR <	1.44	5.80
IL-15	OOR <	OOR <	OOR <
IL-17	OOR <	3.11	9.38
Eotaxin	OOR <	3.13	18.22
Basic FGF	OOR <	4.07	7.99
G-CSF	OOR <	2.12	12.87
GM-CSF	17.29	17.2	24.01
IFN-gamma	OOR <	13.24	45.63
IP-10	OOR <	10.67	19.61
MCP-1 (MCAF)	OOR <	12.39	68.42
MIP-1alfa	OOR <	0.74	1.53
MIP-1beta	OOR <	OOR <	1.01
PDGF-BB	0.50	8.53	27.32
RANTES	OOR <	1.79	5.75
TNF-alfa	OOR <	6.33	20.99
VEGF	OOR <	99.89	546.07

Table S2. Fiber cross section area of tibialis muscle in tumor-bearing mice. Tibialis muscle cross section area of Fast Twitch (FT) IIa, b and non FT fibers in control, G-361 and A2058 tumor-bearing mice. Values as mean ±SEM. *p<0.05, **p<0.01 versus no tumor (Student t-test for G-361, Dunnett's multiple comparisons test following ANOVA for A2058).

Fiber cross section area (μm²)	All fibers	IIa	IIb	Non-IIa/b
No tumor (n=5)	2216 ± 123	1523 ± 138	2602 ± 246	2381 ± 224
G-361 (n=5)	2206 ± 71	1630 ± 68	2590 ± 194	2454 ± 145
No tumor (n=3)	2339 ± 190	1622 ± 123	2794 ± 289	2778 ± 248
A2058 (n=4)	1754* ± 119	1172* ± 80	2249 ± 94	1705** ± 286

Table S3. IL-6 level in sera from lung cancer patient. Level of IL-6 in sera from patients with lung cancer (Norwegian lung cancer biobank) measured using an ELISA assay.

Patient	IL-6 (pg/ml)	SD	Patient	IL-6 (pg/ml)	SD
**1**	5,77	0,41	**23**	1,52	0,19
**2**	2,53	0,25	**24**	11,31	1,11
**3**	3,14	0,11	**25**	1,4	0,29
**4**	4,16	0,26	**26**	1,17	0,06
**5**	3,93	0,63	**27**	4,16	0,26
**6**	25,67	1,52	**28**	4,98	0,11
**7**	2,75	0,3	**29**	0.66	0,16
**8**	5,34	0,45	**30**	6,23	0,34
**9**	0.19	0	**31**	1,52	0,35
**10**	1,01	0,11	**32**	10,61	0,21
**11**	29,33	0,32	**33**	8,23	0,53
**12**	1,98	0,11	**34**	5,31	0,11
**13**	10,61	1,03	**35**	2,53	0,44
**14**	3,7	0,15	**36**	1,2	0,11
**15**	4,29	0,58	**37**	6,17	0,5
**16**	1,85	0,31	**38**	1,98	0,49
**17**	16,98	0,95	**39**	1,27	0,29
**18**	4,49	0,32	**40**	6,14	0,1
**19**	1,91	0	**41**	6,3	0,8
**20**	8,36	0,64	**42**	1,78	0,11
**21**	2,43	0,11	**43**	4,12	0,23
**22**	2,11	0,27	**44**	0.63	0,11

Table S4. Cancer type in patients from the Human Pharmacogenetic Opioid Study (EPOS) included in this work.

Cancer type	%
urological	13.3
hematological	12
prostate	12
pancreatic	6.7
gastrointestinal	18.7
lung	13.3
mesothelial	2.7
liver	1.3
thyroidea	2.7
breast	6.7
melanoma	2.7
sarcoma	1.3
cancer affecting the reproductive organs	1.3
primary tumor of unknown origin	5.3

Table S5. Weight loss of patients from the Norwegian lung cancer biobank, the Edinburgh Cancer Cachexia Cohort (ECCC) Study, and the Human Pharmacogenetic Opioid Study (EPOS), that provided blood samples for the present study.

Weight loss	Lung cancer biobank (n=76)[a]	ECCC (n=127)[b]	EPOS (n=75)[b]
**<0%**	51% (n=39)	15.7% (n=20)	37.3% (n=28)
**0-5%**	15.8% (n=12)	29.2% (n=37)	16% (n=12)
**5-10%**	19.7% (n=15)	21.2% (n=27)	16% (n=12)
**>10%**	13.2% (n=10)	33.9% (n=43)	30.7% (n=23)

[a] 3 months weight loss

[b] 6 months weight loss

REFERENCES

1 Jostock, T. *et al.* Soluble gp130 is the natural inhibitor of soluble interleukin-6 receptor transsignaling responses. *European journal of biochemistry / FEBS* **268**, 160-167 (2001).

2 Fischer, M. *et al.* I. A bioactive designer cytokine for human hematopoietic progenitor cell expansion. *Nature biotechnology* **15**, 142-145, doi:10.1038/nbt0297-142 (1997).

3 Szasz, A. M. *et al.* Cross-validation of survival associated biomarkers in gastric cancer using transcriptomic data of 1,065 patients. *Oncotarget*, doi:10.18632/oncotarget.10337 (2016).

4 Gyorffy, B., Surowiak, P., Budczies, J. & Lanczky, A. Online survival analysis software to assess the prognostic value of biomarkers using transcriptomic data in non-small-cell lung cancer. *PloS one* **8**, e82241, doi:10.1371/journal.pone.0082241 (2013).
